# Operando Microscopy of Photosynthetic Microbial Biohybrids Using Fluorescent Chemical Probes

**DOI:** 10.1021/acselectrochem.5c00517

**Published:** 2026-03-04

**Authors:** Leanne A. Milburn, Evan I. Wroe, Mairi Eyres, Joshua M. Lawrence, Jenny Z. Zhang

**Affiliations:** † Yusuf Hamied Department of Chemistry, 2152 University of Cambridge, Cambridge CB2 1EW, UK; ‡ Department of Biochemistry, University of Cambridge, Cambridge CB2 1QW, UK

**Keywords:** bioelectrochemistry, pH, membrane potential, microenvironments, confocal microscopy, spatiotemporal tracking, chemical dynamics, photosynthetic biofilms

## Abstract

Photosynthetic biohybrids, often in the form of biophotoelectrochemical devices, aim to achieve solar-to-chemical conversion by pairing biotic and abiotic materials to leverage the beneficial attributes of both. Numerous works have highlighted the importance of a well-tuned bio-electrode interface for high biophotoelectrode performance. However, the dynamics of these interfaces in photosynthetic biohybrids are poorly understood, necessitating the development of new tools to disentangle the various (bio)­chemical processes occurring simultaneously. In this work, we construct an *operando* confocal microscopy platform to deeply analyze the dynamics in biofilm morphology, interfacial pH, and membrane potential. These methods assert that light availability is the main driver of microenvironment formation at the interface of a model *Synechocystis*-indium tin oxide bioelectrode. The potential of the electrode only induces a substantial impact outside of the potential range −0.32 to +0.48 V vs. Ag/AgCl at typical current densities. This toolkit provides a foundation for further elucidating interfacial dynamics and rational design of these systems and can be expanded to other bioelectrochemical constructs.

## Graphical abstract

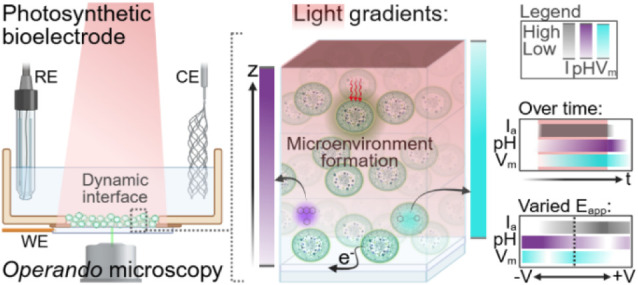

## Introduction

Biotechnologies for sustainable development are rapidly emerging to address various global challenges.^1^ The budding field of semi-artificial photosynthesis aims for sustainable production of energy and energy-dense chemical products by leveraging the combination of biotic and abiotic materials.^2–4^ “Photosynthetic biohybrids” pair microbial cells or enzymes with electrodes or inorganic catalysts, facilitating energy-conversion with only the input of simple starting materials and light energy. This is possible owing to the specificity of biochemical transformations by biocatalysts and the productivity and configurability of artificial materials.^5–9^

While these biohybrids have advanced greatly since their inception, progress toward theoretical efficiencies has proven challenging owing to the dynamic, poorly-understood cell-electrode interactions at the biotic-abiotic interface.^5^ Simultaneous photo-, bio-, and electro-chemistry creates a complex milieu of microenvironments, making performance-limiting factors difficult to identify. For example, a model photosynthetic biohybrid system involves phototrophic microbes (cyanobacteria) interfaced with an electrode for photoelectrochemical water oxidation. Though it is known that light stimulates the photosynthetic electron transport chain (PETC) resulting in extracellular electron transfer (EET),^10^ the cell-electrode interface is still considered a “black box”. Microenvironments could arise from variations in cell density, light intensity, electrochemical potential, and more.^11^ Moreover, they could be dynamic, evolving over time similar to that observed for the EET profile.^12^ Methods to monitor spatial and temporal heterogeneity are important for revealing precise bottlenecks and accelerating rational optimization of this biotechnology.

In recent years, spectroelectrochemistry and *operando* techniques have advanced analysis of the chemical/electrochemical dynamics occurring at the cell-electrode interface in microbial photosynthetic biohybrids.^3,13–15^ However, the morphology of the biofilm remains largely overlooked and spatiotemporally-resolved techniques are crucial to inform on structure-function relationships.^5^ High-resolution microscopy coupled to bioelectrochemistry facilitates such experiments. Confocal fluorescence microscopy, in particular, can effectively resolve microenvironments under operating conditions, as shown for electrode and enzyme-electrode systems.^16–19^

While *operando* microscopy has been used widely in non-photosynthetic biohybrids (e.g., electroactive biofilms) few examples exist for photosynthetic ones. Studies with light-independent biohybrids, using *Shewanella oneidensis*, *Geobacter sulfurreducens*, and others, have provided insight into EET pathways, response to electrical potentials, and environmental dynamics.^20–26^ However, *operando* microscopy on light-dependent constructs is less reported, with only three studies of note. Inglesby *et al.* (2013) performed *in situ* fluorescence microscopy on a dense *Arthrospira maxima* biofilm-electrode biohybrid. This showed differences in cell autofluorescence (and perhaps photosynthetic activity) with proximity to the light source.^27^ Similarly, Liu and Choi (2017) performed *in situ* imaging of a *Synechocystis-Shewanella* co-culture-electrode biohybrid.^28^ They observed biofilm architecture but stopped short of (bio)­chemical insights. Finally, Fu *et al.* (2023) achieved impressive biochemical insights for a photosynthetic *Ralstonia eutropha*-nanoparticle biohybrid by fluorescent-tagging of proteins and subsequently comparing single-cell photocurrent on the microscope.^29^ However, this method is not easily transferable and single-cell analyses miss collective phenomena in microenvironment formation. The importance of microenvironments (including pH effects) have been hypothesized for photosynthetic biohybrids but suitable analytical methods remain scarce.^27^ Especially where phototrophic microbes are applied, existing *operando* fluorescence microscopy methods may be unsuitable as cell autofluorescence limits the choice of fluorophore.

In this work, we addressed this gap by developing *operando* imaging methodologies for photosynthetic biohybrids that reveal overlapping microenvironments at the bio-electrode interface. We employed a model system where cyanobacteria (*Synechocystis* sp. PCC 6803) interface with an indium tin oxide (ITO) electrode (Figure 1A). We hypothesized that microenvironments would emerge in response to light availability and activity at the electrode (Figure 1B). To test this, *operando* confocal microscopy methods were developed for cell mobility (mean square displacement, MSD), extracellular pH (pH_e_), and membrane potential (V_m_)—all presumed to be highly dynamic at the cell-electrode interface (Figure 1C). Cell autofluorescence was leveraged for tracking mobility while fluorescent chemical probes (BCECF and ThT) gave quantitative pH_e_ and V_m_ readouts (Figure 1D). These were tracked in parallel with the photoelectrochemical output (photocurrent) of the biohybrid (Figure 1E,F). We observed dynamic microenvironments at the interface during operational conditions and here identify a suitable potential window for biophotoelectrochemistry, aiding future design of these complex interfaces.

**Figure 1:**
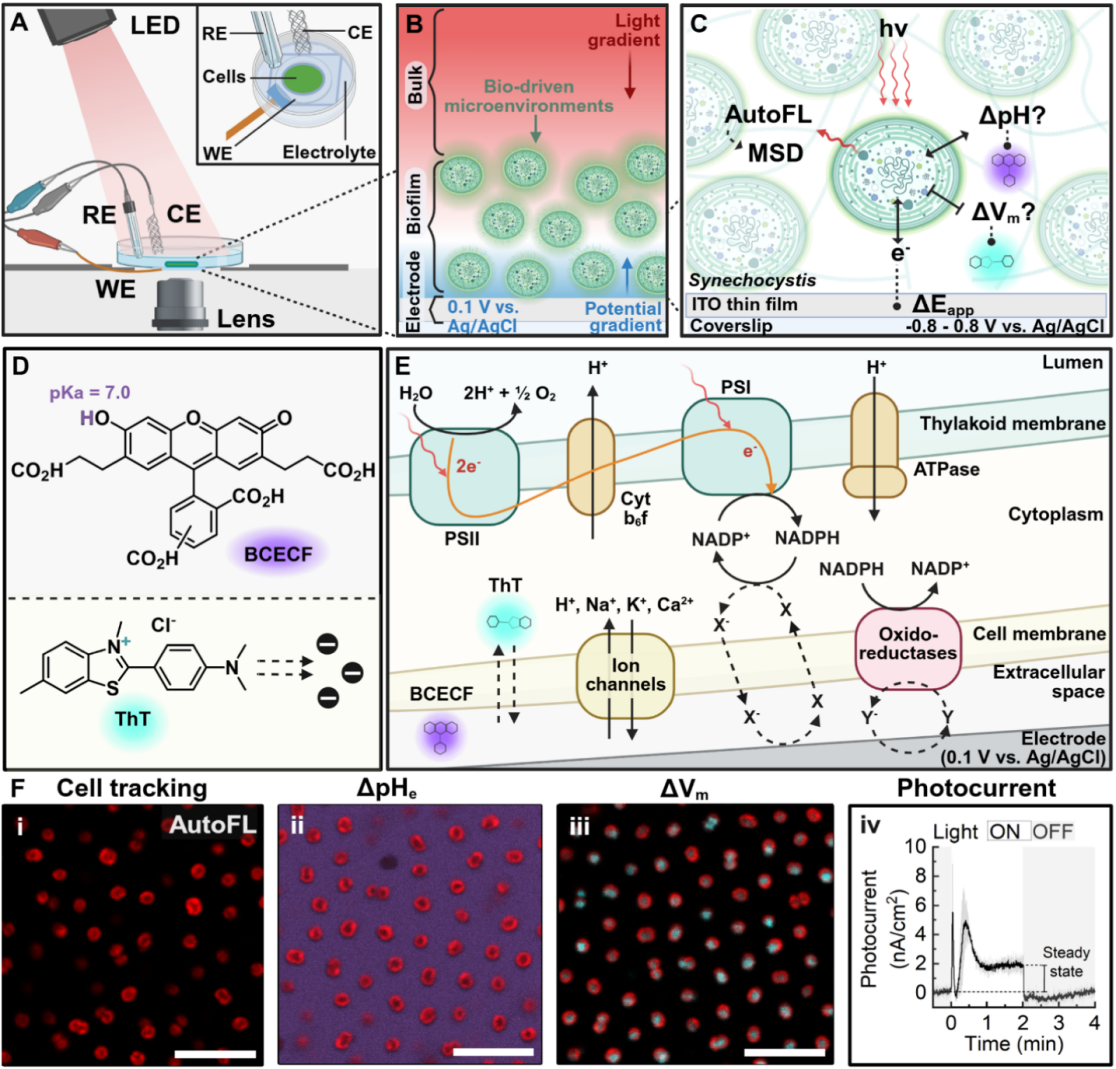
*Operando* confocal microscopy on the *Synechocystis*-electrode interface. (A) Experimental set-up for this work: microscope with actinic (LED) light source and custom, electrochemistry-adapted microscopy dish housing the bioelectrode. Indium tin oxide working electrode (WE), platinum counter electrode (CE), and Ag/AgCl reference electrode (RE). (B) Light (red) and potential (blue) gradients acting on the interface. (C) Magnified view of the interface showing the parameters measured in response to light and applied potential (*E*_app_): cell mean square displacement (MSD) from autofluorescence (AutoFL), extracellular pH (pH_e_) and membrane potential (V_m_). (D) Chemical structures of fluorescent pH (BCECF) and V_m_ (ThT) probes. (E) Localization of probes and tentative molecular mechanism of photocurrent production. (F) Fluorescence confocal images of (i) AutoFL, (ii) BCECF and (iii) ThT with a typical (iv) photocurrent response (scale bars = 10 μm).

## Experimental Section

To enable study of the *Synechocystis*-electrode interface *operando*, a custom electrochemistry-adapted microscopy dish was designed (Figure 1A, Figure S1). In brief, a microscopy dish (ø = 35 mm) was fashioned from polyether ether ketone with a hole (ø = 10 mm) drilled in its center. This served as a well for the cells (V = 200 μL) and an optical window. An ITO-coated glass coverslip (#1.0 thickness, 20 mm^2^, 8−12 Ohms/Sq, sputter coated) was adhered to the underside of the dish, and cells were loaded on top to settle in the dark until use. Once positioned on the confocal laser scanning microscope, the coverslip was connected as a working electrode (SA = 0.785 μm^2^) and a platinum mesh counter electrode and Ag/AgCl reference electrode were suspended into the dish from above. All electrodes were connected to a mobile potentiostat. The electrochemistry-adapted microscopy dish was filled gently with BG11 medium as the electrolyte. Typical BG11 media was used for most experiments; however, a minimal version was utilized when redox-active species from the medium interfered with data interpretation. When used, the fluorescent probes 2′,7′-bis­(2-carboxyethyl)-5-(and-6)-carboxyfluorescein, free acid (BCECF, 5 μM) and thioflavin T (ThT, 10 μM) were added to the electrolyte. 40X oil (NA 1.30) and 20X air (NA 0.65) objective lenses were used for the probes, respectively. Finally, a 680 nm collimated LED was secured onto the microscope stage to act as an actinic light source. A more detailed account of the set-up, procedures, and analysis for each type of experiment can be found in the Supporting Information.

## Results and Discussion

### Biofilm Deposition, Morphology, and Current Output

Cell organization has been proposed to contribute to the magnitude of photocurrent output,^30^ with electrode accessibility and light management being important considerations in biohybrid design. Therefore, we first sought to image different biofilm morphologies and determine the effect on biophotoelectrochemical measurements. A series of cell loading densities were examined, with cells loaded into the electrochemistry-adapted microscopy dish at 1, 10, 25, or 150 nmol_Chl*a*_/mL and left to settle for 4 or 16 h. The resulting biofilm morphology (packing/mobility) and photocurrent were then analyzed. Note that the term “biofilm” is used loosely here as cyanobacteria are not thought to form conventional biofilms.^31^

First, Z-stack imaging was used to probe the packing of the biofilms under different loading conditions (Figure 2A). Quantifying cell count across the image stack revealed that, for loading densities of 10, 25, and 150 nmol_Chl*a*_/mL, cells had organized into distinct layers even after 4 h of settling (Figure 2B). In each case, the bottom layer contained the most cells and each subsequent layer was less populated (Figure 2B, Figures S2, S3). In these conditions, the signal was sufficient for particle-picking up to ∼25 μm into the biofilm, after which noise became prohibitive. With 16 h of settling, the cell layers appeared more loosely packed (increased peak distance) except for the most densely-loaded sample. This may be explained as the secretion of extracellular polymeric substances (EPS) causing a swelling of the biofilm.^31^ Using a loading density of 1 nmol_Chl*a*_/mL produced only one apparent cell layer with relatively sparse surface coverage at the electrode.

**Figure 2:**
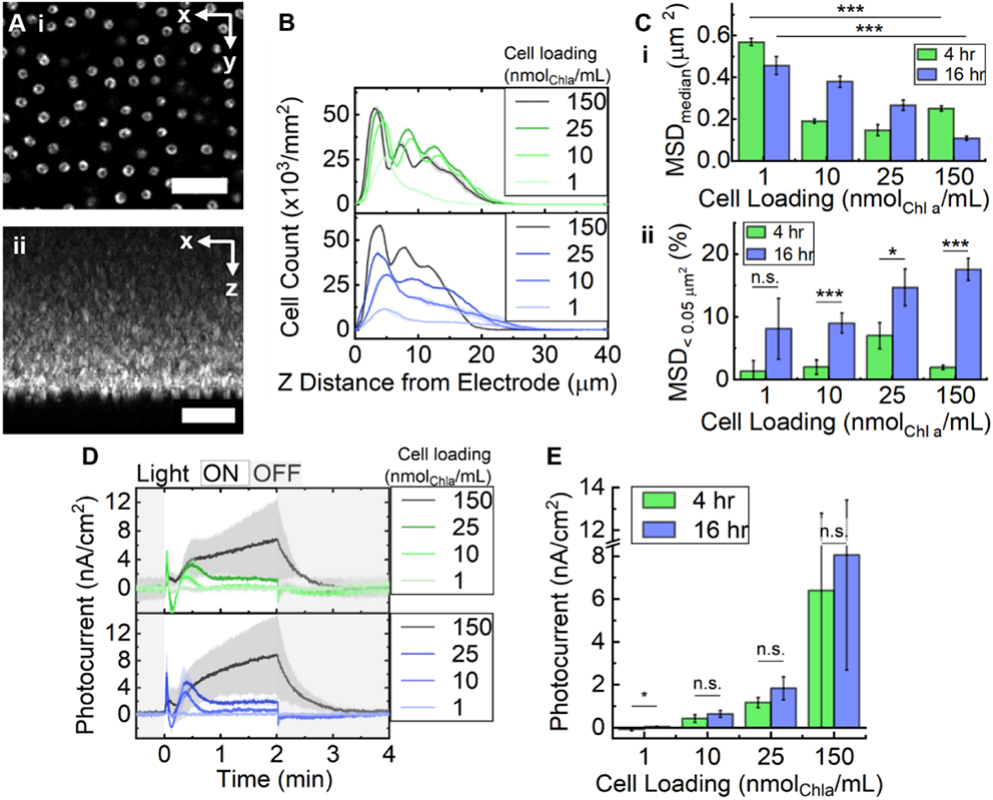
Analysis of *Synechocystis* biofilm morphology when formed with varied cell density and settling time. (A) Representative confocal fluorescence microscopy images of cell autofluorescence showing an x, y view of the cell-electrode interface (i) and the corresponding z-profile (ii). Scale bar = 10 μm. (B) Cell count quantified from z-profile data comparing biofilms settled over 4 or 16 h (green and blue, respectively) with loadings of 1, 10, 25, or 150 nmol_Chl*a*_/mL. (C) Cell mobility analysis quantifying mean square displacement (MSD): median (i) and proportion with MSD < 0.05 μm^2^ (ii) from time-lapse data, same conditions as (B). (D) Photocurrents and (E) steady-state photocurrent magnitude, same conditions as (B) (fifth light/dark cycle shown). Chronoamperometry (E = 0.1 V vs. Ag/AgCl) was done with a three-electrode configuration and light chopping (680 nm, 150 μmol_photons_/m^2^s, 2 min ON/2 min OFF) in BG11 media (pH 7.5). Data shown as the mean and standard deviation (N = 3 biological replicates) with a student’s t-test for pair-wise comparisons (***p < 0.005, *p < 0.05).

Next, we sought to interrogate the behavior of the cell population directly interfacing with the electrode. Cell mobility, described by the MSD, was chosen to assess the stability of the biotic-abiotic interface. A particle-tracking algorithm (Trackpy) was used to determine MSD of single cells in the population during a time-lapse series observing the interfacial cell layer (see workflow in Figure S4). At both settling times, increasing cell loading density resulted in significant decreases in median MSD, suggesting lower mobility and more stability as expected (p < 0.005) (Figure S4; Figure 2Ci). A distinct low-mobility population (MSD < 0.05 μm^2^) was apparent and was, generally, positively correlated with higher cell loading (Figure S4; Figure 2Cii). These are likely cells actively adhered to the electrode surface and thus no longer susceptible to Brownian motion, representing the beginnings of a stable interface. The percentage of surface adhered cells was found to be significantly higher after 16 h compared to after 4 h of settling time (e.g., 9.0% versus 1.9% respectively for the 10 nmol_Chl*a*_/mL condition, p < 0.005).

Finally, wiring of *Synechocystis* to the electrode was assessed by performing chopped-light chronoamperometry. ITO is a common electrode material for use with *Synechocystis* and the photocurrent onset potential is 0.1 V vs. Ag/AgCl when ∼680 nm light is irradiated.^32^ The same conditions are used here, though with a thin-film electrode rather than a structured one, while cycling the actinic light source (150 μmol_photons_/m^2^s, 2 min on/2 min off). The fifth light-dark cycle was used to represent the stabilized system (Figure 2D, Figure S5). Steady-state photocurrent density was calculated as the difference between current at the end of the light period and the preceding dark period. Settling time was found to have no significant effect on photocurrent density in this system (Figure 2E). However, increasing the cell density reliably increased the output photocurrent density. It was notable that the 150 nmol_Chl*a*_/mL samples had a much higher variability in behavior, perhaps owing to variable attachment of cells in the top layers. That photocurrent continues to increase with the addition of cell layers more than ∼25 μm from the electrode further supports the contemporary view that *Synechocystis* exhibits mediated (rather than direct) exoelectrogenic behavior.^30,32,33^

These results present several insights that can inform the design and understanding of *Synechocystis*-electrode biohybrids. More cell layers provide a more stable biofilm and higher photocurrents, but only up to a point. Here, samples loaded at 1 nmol_Chl*a*_/mL of cells formed poorly-adhered biofilms with a negligible photocurrent. 150 nmol_Chl*a*_/mL samples formed thick, adherent biofilms (beyond the depth that we could study), but the photocurrent had a high standard deviation. For further experiments, loading densities of 10−25 nmol_Chl*a*_/mL were used, which gave biofilms with robust cell layers and reproducible photocurrents, and were otherwise similar.

### *Operando* Extracellular pH Measurements with the Fluorescent Probe BCECF

Having assessed biofilm morphology, we next turned to the dynamics of the (bio)­chemical environment. Local pH is a crucial aspect of cell biology and metabolism, with proton availability and translocation influencing cellular activities. In performing biochemical processes, cells may alter their local environment by creating pH gradients and hotspots, which has been previously shown for non-phototrophic biofilms.^34^ The situation is presumed to be even more complex in biohybrid systems where the pH could also be modulated via electrochemistry involving protons/hydroxide ions at the electrode surface.^35^ This has been studied in some enzyme-electrode biohybrid systems^17,36^ but remains poorly understood in microbial biohybrids. pH electrodes are often used for such studies, but the resolution may be limiting and the probe can be invasive.^37,38^ With this in mind, we sought to validate a fluorophore that could elucidate spatiotemporal pH changes in the *Synechocystis*-electrode biohybrid system.

Several commercially-available fluorescent pH probes were screened for use: the pH_e_ probes BCECF and C-SNARF-1, and the intracellular pH (pH_i_) probes BCECF-AM, C-SNARF-1-AM, and acridine orange (Table S3). The pH_i_ probes were deemed unsuitable owing to spectral overlap with cell autofluorescence, non-homogeneous internalization and/or failure to be activated by intracellular esterases (Figures S6−S8). Of the pH_e_ probes, C-SNARF-1 exhibited a poor signal, consistent with its low quantum yield (0.03−0.09) (Figure S7).^39^ However, BCECF boasts a high quantum yield (0.84)^39^ and an absorbance/emission spectrum separate from cell autofluorescence (Figures S6, S9A−C). An extracellular probe is advantageous for investigating both biotic and electrochemical phenomena simultaneously. While pH_e_ of *Synechocystis* cultures in typically measured via pH meter,^40^ this method cannot easily resolve interfacial microenvironments. BCECF is a membrane-impermeable fluorescein derivative which allows quantitative pH measurements, typically by comparing the ratio of the fluorescent signals at 490 and 440 nm confocal laser lines.^39,41^ In this study, we used 488 and 448 nm as these lasers were available on the microscope. BCECF has been used once for pH_e_ studies in microbial biofilms^42^ and also for mammalian cultures^43^ but to the best of our knowledge has not previously been reported in biohybrid systems nor with phototrophic microbes like *Synechocystis*.

BCECF was validated prior to commencing *operando* microscopy experiments. Spectroscopy and fluorimetry confirmed the pH responsiveness of BCECF in BG11 media (Figure S9B,D). Addition of relevant chemical additives (DMSO, DCMU) did not appear to impact the fluorescence of BCECF (Figure S9E,F). Furthermore, BCECF did not impact the vitality of *Synechocystis* as evidenced by a comparable growth curve (Figure S10). Preliminary *operando* microscopy pH analysis demonstrated a clear response to light (increase in fluorescence intensity and BCECF ratio) that was only observed when *Synechocystis* cells were present (Figure 3A, Figure S11A). BCECF alone was photostable under the light regime (Figure S11A) and the probe was confirmed to remain cell-impermeable (Figure S11B). Having sufficiently confirmed BCECF as a reliable pH_e_ probe, a calibration curve was constructed to allow quantitative microscopy experiments (Figure 3B, Figure S12).

**Figure 3:**
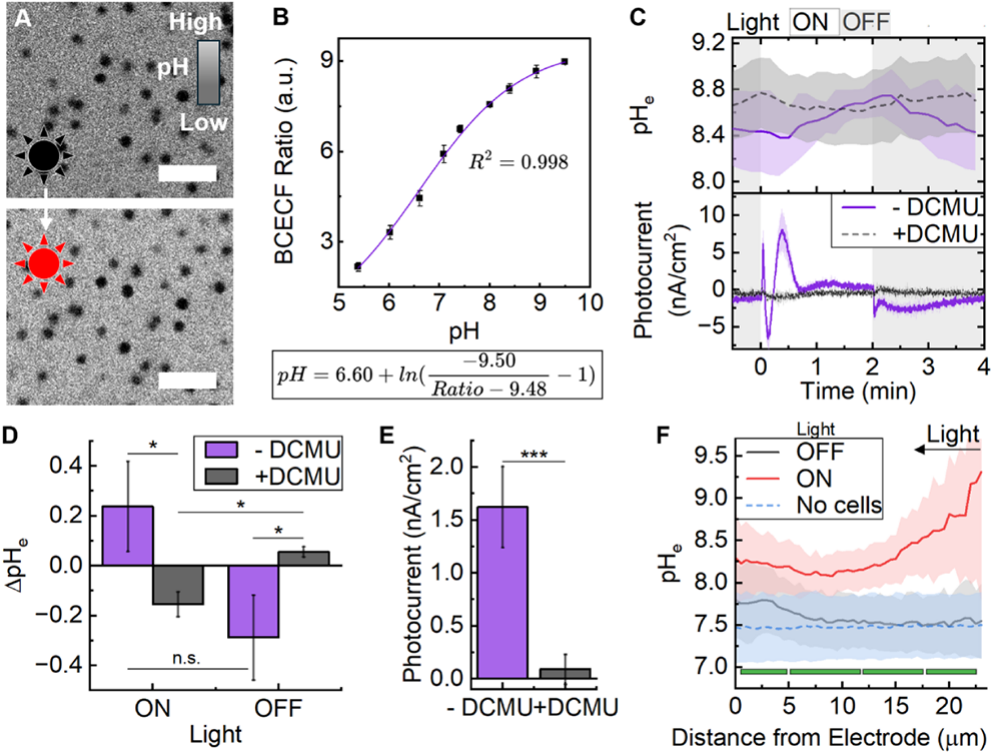
*Operando* pH analysis of a *Synechocystis*-electrode biohybrid using the extracellular pH (pH_e_) probe BCECF. (A) Representative confocal fluorescence images of BCECF at the biohybrid interface before (top) and during (bottom) illumination (680 nm; 150 μmol_photons_/m^2^s). Scale bar = 10 μm. (B) BCECF calibration curve with simplified formula beneath. (C) Simultaneous measurement of pH and photocurrent. Chronoamperometry (E = 0.1 V vs. Ag/AgCl) used a three-electrode configuration and light chopping on a cycle of 2 min on/2 min off in BG11 media (pH 7.5)—fifth cycle shown. Without or with DCMU (50 μM). (D) Quantification of change in pH_e_ and (E) photocurrent from (C). (F) Quantification of pH extending away from the electrode, obtained from Z-stack imaging. Green bars represent the approximate location of cell layers. All data represent the mean and standard deviation (N = 3 biological replicates) with a student’s t-test for pair-wise comparisons (***p < 0.005, *p < 0.05).

*Operando* pH analysis of the biohybrid *Synechocystis*-electrode interface was next performed. *Synechocystis* cells (10 nmol_Chl*a*_/mL) were loaded onto an electrochemistry-adapted microscopy dish and left to settle for 4 h in the dark. BCECF (5 μM) was added in BG11 medium (pH 7.5) and the dish was connected to the potentiostat once on the confocal microscope. The biohybrid was operated by photochronoamperometry, as previously (see Figure 2D). The interface was identified as the bottom-most layer of cells. The fluorescence of BCECF and the photocurrent density were simultaneously recorded over five light/dark cycles, the fifth of which was used to represent the stabilized system (Figure 3C). Initial cycles had alkalinized the extracellular environment substantially, to a pH_e_ ∼8.4. Within the fifth cycle, illumination resulted in a further alkalinization of 0.24 ± 0.18 pH units over 2 min, which reversed completely in the subsequent dark period (Figure 3D). Meanwhile, a typical photocurrent profile was observed with a steady-state value of 1.62 ± 0.38 nA/cm^2^ (Figure 3E).

Cyanobacteria are generally alkalophilic and capable of alkalinizing their external environment through several mechanisms, some of which are photosynthetic. CO_2_ uptake and processing has been proposed to result in the export of hydroxide ions into the extracellular microenvironment.^44–46^ However, pH regulation at the plasma membrane of *Synechocystis* is complex and not yet fully understood. Na^+^/H^+^ antiporters have been shown to influence pH_e_ response with light-induced alkalinization attributed to H^+^ uptake which exerts a positive impact on the PETC.^40^ Conversely, a transient acidification of pH_e_ has been attributed to H^+^ export by proton exchangers (e.g., PxcA), thought to aid the formation of proton motive force at the PETC.^47^ To verify whether pH_e_ and the PETC were correlated at the interfacial microenvironment of our biohybrid, we added the photosystem II inhibitor DCMU (50 μM). DCMU abolished the light-triggered ΔpH_e_ (−0.16 ± 0.05) and photocurrent (0.09 ± 0.14 nA/cm^2^), significantly decreasing both (p < 0.05, p < 0.005) and suggesting that pH_e_ is dependent on photosystem II activity (Figure 3C−E). Further, cells killed by heat-treatment produced no fluctuations, confirming that active metabolism is a necessity (Figure S13).

It was interesting to note that the ion-flux-based biotic pH_e_ fluctuations seen in bulk systems were also occurring at the interface of the biohybrid device. In other biohybrid devices, pH has been proposed to fluctuate due to pH-coupled reactions at the electrode surface, with acidification arising from oxidation of electron donors.^36^ However, we found no evidence of this, instead seeing alkalinization during photocurrent generation. To further this study, pH_e_ was compared at the surface of the electrode and extending away from it while the system was held at 0.1 V vs. Ag/AgCl (Figure 3F). Before illumination, confocal Z-stack images showed a relatively consistent pH_e_ across a 25 μm section from the electrode into the upper cell layers of the biofilm. During illumination, the pH_e_ increased more at the upper layers of cells than at the electrode surface, with a positive slope of the trace only being observed at upper layers. While this could be evidence of proton-coupled electron transfer counteracting the biotic alkalinization, it is more likely that the biotic effect is more pronounced in cell layers with greater illumination (less shaded). This was further supported by the observation that pH_e_ fluctuations were of a similar magnitude in the absence of an electrode (Figure S11A). These results suggest that pH_e_ is primarily governed by biotic processes in *Synechocystis*-ITO electrode biohybrids, at least while current density is low. This implies that is the endogenous EET mediator of *Synechocystis* is either not proton-coupled or (more likely) too dilute to be measured here. Situations where current density is higher (e.g., when using proton-coupled exogenous mediators such as quinones) remain to be studied. Furthermore, the variability of light exposure throughout the biofilm creates a gradient of pH_e_ microenvironments (Figure 1B) that may impact overall biohybrid performance, for example, by differentially regulating gene expression throughout the *Synechocystis* biofilm.^48^

### Membrane Potential Dynamics Tracked Using the Nernstian Dye Thioflavin T

As pH is fundamentally intertwined with V_m_, elucidating V_m_ dynamics adds depth to the pH_e_ study above. V_m_ is the difference in electrical charge across a lipid membrane, arising from several dynamic biochemical phenomena. These include the distribution of ions (predominantly K^+^, Na^+^, and Ca^2+^), pH gradients, ATP synthesis, electron transport, and the permanent distribution of charged species (e.g., DNA, RNA, charged phospholipids).^49^ Recent studies have proposed V_m_ as a means of coordinating diverse functions across the bacterial community, such as biofilm growth,^50^ cell signaling,^51^ memory,^52^ and response to external stimuli.^53,54^ In cyanobacteria, *Oscillatoria* was recently shown to engage in Ca^2+^ signaling linked to current spiking behavior.^55^ This previous work suggests that V_m_ should be highly dynamic in the *Synechocystis*-electrode biohybrid system.

Nernstian fluorescent probes are plasma membrane-permeable, cationic molecules that diffuse across the membrane according to V_m_. In this way, assuming negligible extracellular fluorescence, intracellular probe fluorescence can be utilized as a proxy for V_m_. In recent years, ThT has been applied to study V_m_ in several bacterial biofilms and biohybrids.^50–54^ ThT is known to respond rapidly and is thus a good candidate for *operando* V_m_ studies.^56^ As the cell interior becomes more negative (hyperpolarization), intracellular ThT fluorescence should increase, as the positive ThT molecules diffuse into the cell. Oppositely, a decrease in intracellular ThT fluorescence denotes a more positive interior (depolarization). This probe has yet to be tested in phototrophic microbes such as *Synechocystis*.

First, the ability of ThT to act as a Nernstian probe in *Synechocystis* was tested, as per the workflow of Mancini *et al.* (2020).^57^ ThT has a fluorescence spectrum compatible with *Synechocystis* autofluorescence (Figure S14), making it preferable over the red-emitting Nernstian probes (e.g., TMRM, DiSC_3_, Di-4-ANEPPS). 10 μM ThT is a standard concentration^50,54^ and enabled use of low laser intensities on the confocal microscope, minimizing cell disturbances. While this concentration negatively impacted *Synechocystis* cell growth (over days-weeks), the rate of photosynthesis (O_2_ evolution) and photocurrent were unaffected, so short-term use (<1 h) was deemed acceptable (Figure S15). Initial high-resolution imaging confirmed cytosolic probe localization, with internalization kinetics suggesting diffusion rather than active transport (Figure 4A,B and Figure S16). Addition of the protonophore CCCP (1 mM) was found to abolish V_m_ and cause the dissolution of intracellular ThT fluorescence (p < 0.005) (Figure 4C). Further, addition of the K^+^ ionophore valinomycin increased intracellular ThT fluorescence, while subsequent additions of KCl (1−500 mM) reduced it again. This demonstrated a dynamic ΔV_m_ range of −85 to +15 mV and a limit of detection on the order of ∼1 mV (Supplementary Note 1, Figure S17). Tests were conducted on whether ThT could be calibrated to give quantitative values; however, these proved to be unreliable and qualitative assessment was deemed as sufficiently informative.

**Figure 4:**
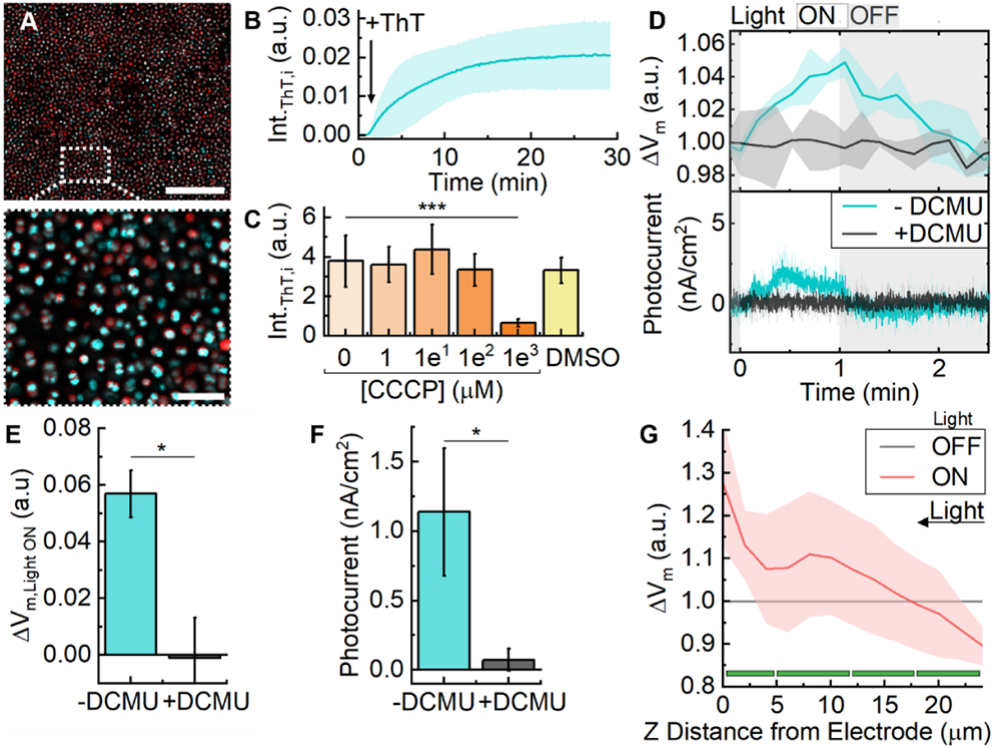
*Operando* analysis of membrane potential changes (ΔV_m_) in a *Synechocystis*-electrode biohybrid using the fluorescent probe ThT. (A) Representative confocal fluorescence images of *Synechocystis* stained with ThT at the biohybrid interface. Scale bar = 20 μm (top) and 10 μm (bottom). (B) Mean intracellular ThT fluorescence intensity following ThT addition (10 μM; arrow). (C) Mean intracellular ThT fluorescence intensity following addition of various concentrations of CCCP dissolved in 1% (v/v) DMSO, along with a DMSO control. (D) Simultaneous measurement of the ΔV_m_ and photocurrent. Chronoamperometry (E = 0.1 V vs. Ag/AgCl) used a three-electrode configuration and light chopping (680 nm, 150 μmol_photons_/m^2^s) on a cycle of 1 min on/1.5 min off in BG11 media (pH 8.5)—fifth cycle shown. Without or with DCMU (200 μM). (E) Comparison of ΔV_m_ and (F) current density during the light period from D. (G) ΔV_m_ in the biofilm extending away from the electrode (0.1 V vs. Ag/AgCl) during illumination, relative to the preceding dark period. Green bars represent the approximate location of cell layers. ΔV_m_ qualitatively represented by ThT fluorescence in all data (Methods). Data presented as the mean and standard deviation (N = 3 biological replicates). A student’s t-test was used for pair-wise statistical comparisons (***p < 0.005, *p < 0.05).

Having validated ThT for use in *Synechocystis*, *operando* studies of the change in cellular membrane potential (ΔV_m_) were performed. *Synechocystis*-electrode biohybrids were constructed as described before, with slight differences in cell loading (25 nmol_Chl*a*_/mL, 16 h settling) but yielding a similar biofilm to those used for pH studies (see Figure 2). ThT (10 μM) in BG11 (pH 8.5) was added to the dish and equilibrated for 20 min. On the microscope, the biohybrid was again subjected to photochronoamperometry with photocurrent simultaneously recorded (Figure 4D, light cycling 1 min on/1.5 min off, fifth cycle plotted). At the bottom-most layer of cells, ThT fluorescence showed that V_m_ hyperpolarized over 1 min of light exposure and returned to baseline again during the dark (Figure 4E). As before, a typical photocurrent profile was observed due to light-driven EET with a steady-state value of 1.14 ± 0.37 nA/cm^2^ (Figure 4D,F).

These results corroborate previous studies showing a light-induced hyperpolarization in V_m_ in cyanobacteria.^58,59^ Addition of DCMU (200 μM) significantly inhibited the ΔV_m_ and photocurrent (0.07 ± 0.07 nA/cm^2^, p < 0.05), confirming that the ΔV_m_ was photosynthetic in origin (Figure 4C−F). This ΔV_m_ activity likely resulted from proton pumping across the thylakoid membrane during photosynthesis, causing a relative hyperpolarization of the cytosol (where ThT is located, see Figure S16).^59,60^ However, we cannot exclude the possibility of a contribution from light-induced ion transport at the plasma membrane. Interestingly, though, this trend in ΔV_m_ is counter to the trend in pH_e_: biotic extracellular alkalinization alone would cause V_m_ to depolarize as protons enter (or hydroxide ions exit) the cytosol. Either the extracellular alkalinization is charge-balanced, perhaps again relating to Na^+^/H^+^ antiport at the plasma membrane,^40,61^ or the ΔV_m_ at the thylakoids dominates over that at the plasma membrane. To further investigate this dynamic and determine whether V_m_ was stratified according to light availability or electrode proximity, we studied V_m_ as a function of distance from the electrode. As before, cells were held at 0.1 V vs. Ag/AgCl and Z-stacks 25 μm in depth were collected before and during illumination. Here, ΔV_m_ was calculated such that each slice in the illuminated condition was relative to the corresponding slice from the dark condition. From our pH studies, we expected to see a larger ΔV_m_ in the less-shaded, upper cell layers but the electric field from the electrode could also play a role. At lower layers of the biofilm, cells hyperpolarized during light exposure, consistent with the bottom-most layer (Figure 4G). However, V_m_ depolarized in the upper layers following illumination. Where there is more light exposure, extracellular alkalinization presumably has a greater impact on V_m_, which may explain why pH_e_ and V_m_ results support each other at upper cell layers but not at lower, shaded ones (Figures 3F, 4G). Again, the electric field did not appear to have influence; ThT fluorescence increased toward the interface, opposite to what would be expected if negative charges (electrons) were being drawn out of the cells.

Evidently, at this mild potential (0.1 V vs. Ag/AgCl), which is close to the open circuit potential of the system, the electrode is not contributing significantly to the biofilm’s behavior.^32^ To investigate at what point the electrode did have influence, we proceeded to test the effect of a large range of potentials on the biofilm environment.

### Effect of Extreme Potentials on the Biofilm Environment

In the previous sections, we have demonstrated methods to measure cell mobility, pH_e_, and V_m_. This data suggests that a gradient in light availability, rather than an electrical gradient, is driving the formation of microenvironments in the biohybrid. Since our experiments were performed under a mild oxidizing environment (0.1 V vs. Ag/AgCl) we next questioned whether extreme applied potentials (*E*_app_) would induce changes in cell mobility, pH_e_, and V_m_. Typical studies of cyanobacteria-electrode biohybrids apply mild conditions (0.1−0.3 V vs. Ag/AgCl),^33,62,63^ however, more forcing conditions are not uncommon (−0.4 V or 0.5−0.7 V vs. Ag/AgCl).^64–67^ The impact of these different potentials has not yet been studied. Here, we omitted the actinic light so that only changes due to potential effects would be observed. Initial control scans revealed that the transition metals in BG11 convoluted the voltammograms (Figure S18), so a minimal BG11 medium was used as the electrolyte for all following experiments (see Methods). Cells were washed three times with this medium prior to loading.

First, the *Synechocystis*-electrode biohybrids were assessed for cell mobility at extreme potentials. 5 min time-lapses of the bottom-most layer of cells were collected at 0.0, +0.8, and −0.8 V vs. Ag/AgCl on the confocal microscope, representing the extreme ends of a cyclic voltammogram. Cell mobility was analyzed, extracting the median MSD and proportion of adhered cells as before (Figure S19, Figure 5A). We found that a very positive potential (+0.8 V) had no impact on cell mobility, however a high negative potential (−0.8 V), appeared to “shock” the cells, inducing a significant decrease in median MSD and a significant increase in low-mobility cells (p < 0.01, p < 0.05; Figure 5A). This behavior was reversible, with cells recovering to near-baseline mobility after returning to a moderate *E*_app_.

**Figure 5:**
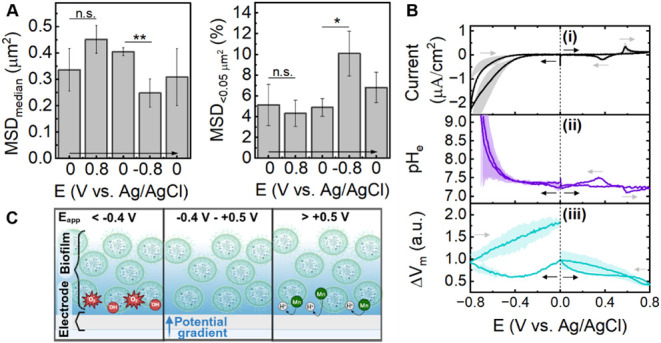
Impact of applied potentials (*E*_app_) on the *Synechocystis*-electrode biohybrid in the dark. (A) Cell mobility analysis showing the mean square displacement (MSD) from time-lapse images at held the noted *E*_app_ in the sequence indicated. Left, cell population median; right, proportion of low-mobility cells (MSD < 0.05 μm^2^. (B) Cyclic voltammetry (i) with *operando* extracellular pH (pH_e_; ii) and change in membrane potential (ΔV_m_; iii) analysis using the fluorescent probes BCECF and ThT. Scan rate 1 mV/s; cathodic and anodic scans performed on separate samples; starting from 0 V. A three-electrode configuration was used (indium tin oxide working, platinum mesh counter, Ag/AgCl reference electrodes, in minimal BG11 media). (C) Schematic illustrating the chemical effects of extreme *E*_app_ on the biohybrid. All data are presented as the mean and standard deviation (N = 3 biological replicates). A student’s paired t-test was used for pair-wise statistical comparisons (**p < 0.01, *p < 0.05).

Next, the biohybrids were paired with BCECF or ThT, as described above, and cyclic voltammetry (CV) studies were performed. Voltammograms with a scan rate of 1 mV/s were conducted between 0.0 and −0.8 V or 0.0 and +0.8 V vs. Ag/AgCl, with different samples used for each (Figure 5B). Under mild applied potentials (0.1−0.3 V vs. Ag/AgCl) used in this paper and others,^33,62,63^ no change in pH or V_m_ was observed (in darkness). This confirmed that the electric field should have had a minimal effect in the previous results. As expected, sweeping from 0.0 V to potentials more negative than −0.32 V induced a strong reductive peak indicative of O_2_ reduction (Figure 5Bi). O_2_-free controls confirmed O_2_ involvement (Figure S20) and while ITO reduction is possible, it is likely disfavored under our experimental conditions.^68^ This cathodic peak coincided with an increase in pH_e_, suggesting generation of hydroxide ions during O_2_ reduction pathways (Figure 5Bii).^69,70^ This could also explain the cells being “shocked” into low-mobility states, if cellular energy was being reallocated to maintaining homeostasis rather than motility (Figure 5A). V_m_ did not respond in as simple a manner. Rather, depolarization was observed at low negative potentials but irreversible hyperpolarization occurred after ∼−0.4 V, with a continued rise on the reverse sweep (Figure 5Biii). This apparent dysbiosis could be a response to ROS accumulation, which was not directly measured here. However, cell membrane integrity appeared to remain intact as evidenced by continued exclusion of BCECF from the intracellular space (Figure S21). Interestingly, in the positive potentials, a reversible CV wave was observed with a midpoint potential (E_m_) of +0.48 V (Figure 5Bi). Parallel pH_e_ measurements suggest that this reaction is pH-coupled, with the anodic peak causing acidification and the cathodic peak causing re-alkalinization (Figure 5Bii). This pH change was not observed in the abiotic control, indicating that it arises from a species secreted by, or bound to, the cells (Figure S22A). The origin of this CV peak is unclear, but one explanation is the proton-coupled oxidation of manganese chloride which has been observed with *Synechocystis* before.^63^ Abiotic CV confirmed that oxidation of Mn^2+^ complexes onsets around +0.48 V under our experimental conditions (Figure S23). Manganese species are perhaps retained in the EPS of *Synechocystis* and/or secreted from live or dead cells. Here, V_m_ responded to the electrochemical changes as a steady hyperpolarization occurred up to ∼+0.5 V in the positive sweep (Figure 5Biii). The steady decline may be explained by the oxidizing potential drawing electrons out of the cells, similar to observations with other species^54^ and consistent with the higher photocurrents observed in biohybrids using more oxidizing biases.^32,63^ At extreme oxidizing potentials (∼+0.7 V), V_m_ appears to drop more steeply, however, an additional oxidation peak on the CV spectrum was also observed (Figure S22B). This suggested that ThT itself was starting to be oxidized and should not be used above these potentials.

Overall, these results show that the *Synechocystis* biofilm is relatively resilient against the impacts of mild applied potentials. However, potentials below −0.32 V vs. Ag/AgCl (on an ITO working electrode) should be used cautiously as O_2_ reduction becomes prominent after this point. Further, potentials above +0.48 V vs. Ag/AgCl induce redox, pH_e_, and V_m_ changes within the biofilm that may impact cell behavior and the mechanism of biohybrid function. The importance of the biofilm-localized redox species observed at +0.48 V has yet to be properly investigated. A suitable potential window for *Synechocystis*-ITO electrode biohybrids is therefore suggested as −0.32 to +0.48 V vs. Ag/AgCl to allow for consistent interpretation of results.

## Conclusions

In this work, we have developed a robust platform for obtaining structural and (bio)­chemical dynamics alongside electrochemical readout in a functional photosynthetic biohybrid system. Applying this to study the *Synechocystis*-ITO electrode interface, we can deconvolute the role of multiple intersecting processes.

Firstly, the morphology of the biofilm can dramatically change the behavior of the biohybrid, dictating both the diffusivity of cells in the biofilm and photocurrent output. We found that increased settling time caused cell−cell packing to decrease, presumably due to EPS secretion. Higher cell loading increased photocurrent production through the contribution of distal cell layers, supporting the case for a diffusible mediator. Secondly, BCECF and ThT were found to be useful fluorescent probes for cyanobacterial systems as trackers of pH_e_ and V_m_, respectively. BCECF use supported the understanding of cyanobacterial biofilms regulating their microenvironments, alkalinizing the extracellular space reversibly during biohybrid operation (illumination). pH_e_ increased more in upper cell layers suggesting that light availability (photosynthesis) dictates pH_e_, rather than redox reactions at the electrode, as depicted in Figure 1B. ThT was demonstrated as a viable and sensitive qualitative probe of membrane potential changes in *Synechocystis*. ΔV_m_ measurements showcased the balance of metabolic processes in varied environments, with thylakoid proton-pumping appearing dominant in shaded cell populations while plasma membrane ion (e.g., H^+^) pumping became dominant in unshaded ones. Together, BCECF and ThT revealed that light availability is the principle source of microenvironment formation in this *Synechocystis*-electrode system, at least at low current densities. Lastly, BCECF and ThT were applied to understand which electrode conditions would induce (bio)­chemical changes to the interface. While plasma membrane integrity held up to the studied potential range (−0.8 to +0.8 V vs. Ag/AgCl), evidence of O_2_ reduction and biofilm-localized redox reactions suggests a useful operating window of −0.32 to +0.48 V vs. Ag/AgCl within which the electrode should not be convoluting the mechanism of electron transfer.

Overall, our work highlights the complexity of photosynthetic biohybrid systems, particularly at the interface. However, the developed methodology can help to disentangle several simultaneous processes—elucidating biofilm morphology, pH_e_, and V_m_ at the cell-electrode interface and revealing the role of light gradients and electrode potentials in this model system. This toolkit can be easily transferred to the study of similar systems, for example, investigating the role of electrode architecture or the impact of exogenous electron mediators. More broadly, we can envision our *operando* microscopy methods being applied to a variety of microbial and non-microbial bioelectrochemical and biohybrid systems to better understand how (bio)­chemical dynamics influence electrochemical outputs.

## Supplementary Material



## Data Availability

All raw experimental files and metadata are openly available in the Cambridge Research Repository, Apollo (CC BY 4.0 licence): DOI 10.17863/CAM.127863.

## Notes

Detailed materials and methods and additional supporting data (Figures S1−S24, Tables S1−S3) (PDF)
