# Supplementary material for: Operando Microscopy of Photosynthetic Microbial Biohybrids Using Fluorescent Chemical Probes
Source: ACS Electrochem. 2026 Mar 4;2(4):964–73. doi: 10.1021/acselectrochem.5c00517 (PMC13051432; doi:10.1021/acselectrochem.5c00517)
Supplement: Supplementary file 1 [file ec5c00517_si_001.pdf]

Supporting Information for:

Operando Microscopy of Photosynthetic Microbial Biohybrids using Fluorescent Chemical Probes

Leanne A. Milburn[1†], Evan I. Wroe[1†], Mairi Eyres[1], Joshua M. Lawrence[1,2], Jenny Z. Zhang[1*]

1. Yusuf Hamied Department of Chemistry, University of Cambridge, Cambridge, UK, CB2 1EW
2. Department of Biochemistry, University of Cambridge, Cambridge, UK, CB2 1QW

† These authors contributed equally

Corresponding author: Jenny Z. Zhang, Yusuf Hamied Department of Chemistry, University of Cambridge, Cambridge, UK, CB2 1EW, phone +44 7599844982, jz366@cam.ac.uk

Table of Contents

Materials and Methods 3

Materials 3

Media for Cell Culture and Electrolyte 3

Cell Culturing 4

Preparing Cells for Biohybrid Assembly 4

Assembly of Electrochemistry-Adapted Microscopy Plates 4

Fluorescent Probe Preparation 5

Operando Confocal Microscopy on the Synechocystis-Electrode Biohybrid Constructs 5

Operando pH Measurements with BCECF 5

Operando Membrane Potential Measurements with ThT 7

Fluorimetry 8

Oxygen Evolution 8

Statistics 8

Figure S1. 9

Figure S2. 10

Figure S3. 11

Figure S4. 12

Figure S5. 13

Table S3. 14

Figure S6. 15

Figure S7. 16

Figure S8. 17

Figure S9. 18

Figure S10. 19

Figure S11. 20

Figure S12. 21

Figure S13 22

Figure S14. 22

Figure S15. 23

Figure S16. 24

Figure S17 25

Figure S18. 26

Figure S19. 26

Figure S20. 27
Figure S21. 27
Figure S22. 28
Figure S23. 28
Supplementary Note 1 29
Figure S24 30
References: 31

Materials and Methods

Materials

All standard reagents were obtained from commercial suppliers and used as received unless otherwise noted. Thermo Fisher Scientific was used to source acridine orange (AO; Invitrogen), 5-(and-6)-carboxy SNARF™-1, Acetoxymethyl Ester, Acetate (SNARF AM; Invitrogen), 5-(and-6)-carboxy SNARF™-1 (SNARF; Invitrogen), 2',7'-bis(2-carboxyethyl)-5-(and-6)-Carboxyfluorescein, Acetoxymethyl Ester (BCECF AM; Invitrogen), 2',7'-bis(2-carboxyethyl)-5-(and-6)-Carboxyfluorescein (BCECF Free Acid; Invitrogen), Thioflavin T (ThT), and Fluorescein Isothiocyanate-Dextran (FITC-Dextran). All fluorescent probes were dissolved in DMSO (except ThT, which was dissolved in water) and stored as frozen stock solutions until use. All aqueous solutions were prepared with mQ water (<18.2 MΩ•cm).

An Ivium PocketStat2 mobile potentiostat was used for all electrochemical measurements along with the IviumSoft software. Indium tin oxide (ITO)-coated coverslips (#1.0 thickness, 20-22 mm^2, 8-12 Ohm/Sq) were purchased from Diamond Coatings Ltd.. A BASi Ag/AgCl (3M NaCl) reference electrode was used. The actinic 680 nm light emitting diode (LED) and collimator were obtained from Thorlabs. The custom-designed polyether ether ketone microscopy dishes were prepared by the Mechanical Workshop at the Yusuf Hamied Department of Chemistry.

Media for Cell Culture and Electrolyte

BG11 media (pH 7.5) was used to grow cells as described below. This media was formulated according to Rippka et al. (1979).[1] This recipe was deemed appropriate as it yields a near-neutral solution (pH 7.5) and omits supplemental buffering species. This allowed pH to be studied within the accurate range of the fluorescent reporter BCECF.

BG11: $NaNO_3$ (1.5 g/L), $K_2HPO_4{\cdot}3H_2O$ (0.04 g/L), $MgSO_4{\cdot}7H_2O$ (0.075 g/L), $CaCl_2{\cdot}2H_2O$ (0.036 g/L), citric acid (0.006 g/L), ferric ammonium citrate (0.006 g/L), Na_2EDTA (0.001 g/L), Na_2CO_3 (0.02 g/L). To this, trace minerals mix was added (1 mL per litre of BG11) : H_3BO_3 (2.86 g/L); $MnCl_2{\cdot}4H_20$ (1.81 g/L); $ZnSO_4{\cdot}7H_2O$ (0.222 g/L), $Na_2MoO_4{\cdot}2H_2O$ (0.390 g/L), $CuSO_4{\cdot}5H_2O$ (0.079 g/L), $Co(NO_3)_2{\cdot}6H_20$ (0.0494 g/L)

A minimal BG11 media was used when necessary to avoid convoluting signals in electrochemical characterisation (e.g. redox peaks from manganese). This minimal media was informed by the work of Yang *et al.* (2015) which showed minimal growth effects on excluding BG11 components except for a few.[2]

Minimal BG11: $NaNO_3$ (1.5 g/L), $K_2HPO_4{\cdot}3H_2O$ (0.04 g/L), $MgSO_4{\cdot}7H_2O$ (0.075 g/L)

Cell Culturing

Wild-type *Synechocystis* sp. PCC 6803 stocks were purchased from the Pasteur Institute, France, and streaked on BG11 agar plates to culture until single colonies formed. Colonies were transferred to liquid BG11 media in Nunc flasks. Liquid cultures were grown under white light (ca. 40 µmol photon/m^2s, 30C, shaking). and harvested at an OD_{750} of 1.0 +/- 0.1 for experiments. Chlorophyll quantification was done via Equation 1, with absorbance measurements on a UV-Vis spectrophotometer (Agilent Tech Cary 60) using BG11 media as the solvent.

$$[\text{Chlorophyll } a] \text{ (nmol/mL)} = (A_{680} - A_{750}) \times 10.854$$

Equation 1.[3] Chlorophyll *a* quantification in cyanobacteria.

Preparing Cells for Biohybrid Assembly

An aliquot of *Synechocystis* cells (OD_{750} = 1.0 +/- 0.1) was removed from the culture flask and centrifuged for 2 minutes at 3000 x g to pellet the cells. The pellet was redispersed in fresh BG-11 medium (pH 7.5 for pH work or pH 8.5 for ThT work) to obtain the required concentration (1, 10, 25, or 150 $nmol_{Chl a}$/mL). 200 µL of this solution was plated onto the custom-built microscopy plate (see below) and left to form a biofilm in the dark for 4 or 16 hours, in a closed, damp chamber to prevent water evaporation.

Assembly of Electrochemistry-Adapted Microscopy Plates

The custom three-electrode electrochemical microscopy dish was constructed as follows. To an ITO-coated glass coverslip, a 5 cm piece of copper tape was secured such that the conductive sides of both components were intimately contacting. A custom, reusable polyether ether ketone (PEEK) microscopy dish was manufactured in-house, with a ø = 10 mm hole in its centre. The ITO-coverslip was secured to the underside of the PEEK microscopy dish using a hydrophobic, non-toxic adhesive (either Blu Tack or nail varnish). In preparation for use, cells were deposited in the central well and left to settle (for 4 or 16 hours), and electrolyte (BG11 or mBG11 containing the fluorescent probe of interest) was added immediately before starting experimentation.

For electrochemistry experiments, a platinum mesh counter electrode and Ag/AgCl reference electrode were suspended in the BG-11 medium, secured using electrical tape and Blu-Tack. Electrodes were connected to a mobile potentiostat, used in conjunction with the IviumSoft software for data collection. When electrochemistry was not required, a non-conductive (#1.5 glass) coverslip was instead used, without copper tape attached.

Fluorescent Probe Preparation

All fluorescent probes (received as powder) besides ThT were dissolved in DMSO and aliquots were stored as a concentrated (5 mM) stock solution at -20˚C whenever not in use. ThT was dissolved in water and stocks (10 mM) stored at -20˚C. All solutions were protected from light. Stocks were diluted in BG-11 prior to use. The fluorescent probes were added to final concentrations of 5 µM (BCECF) or 10 µM (ThT) such that the final concentration of DMSO was less than 1 % (v/v). Treated samples were given 5 minutes (BCECF) or 20 minutes (ThT) to equilibrate prior to starting experimentation.

Operando Confocal Microscopy on the Synechocystis-Electrode Biohybrid Constructs

Confocal fluorescence microscopy was carried out using a Leica Stellaris 5 Confocal Microscope, available through the Molecular Production and Characterisation Centre in the Yusuf Hamied Department of Chemistry. This microscope was equipped with three laser lines (405 nm and 448 nm diode laser and 488 nm solid state laser) and an integrated white light laser, tuneable to wavelengths between 485 – 685 nm in 1 nm intervals. Four HyD S detector channels were available for up to four-colour imaging, and two HC PL APO CS2 objectives were used (20X, NA 0.75, dry and 40X, NA 1.30, oil). Exact imaging conditions for each fluorescent probe are detailed below.

The sample plate, as described above, was set up on the confocal microscope. 3.5 mL of BG-11 was added to the plate dropwise, so as not to disturb the biofilm. The fluorescent probe of interest was pre-mixed into this volume, as was any additional component (e.g., DCMU). A collimated actinic LED (680 nm, Thorlabs) was secured above the sample on the viewing platform and focused on the biofilm (150 µmol photon/m^{2}s).

Electrochemical readout from the biohybrid was facilitated by poising the working electrode at 0.1 V vs. Ag/AgCl (chronoamperometry mode). A current range of 10 nA was used with sampling every 0.1 or 0.2 s. Each sample was pre-equilibrated to a stable current baseline prior to data collection. Photocurrents were calculated as the difference between the steady-state current in the light versus the dark before. The fifth light/dark cycle was used for this calculation.

When cyclic voltammetry was performed to assess the effect of applied potential ranges, separate samples were used for the positive (0-0.8 V vs. Ag/AgCl) and negative (0-(-0.8) V vs. Ag/AgCl) scan directions, with a current range of 1 µA, a scan rate of 1 mV/s, and sampling every 1 s.

Operando pH Measurements with BCECF

Light chopping was performed on a cycle of 2 minutes ON/2 minutes OFF starting 30 seconds after the time-lapse was initiated. Experiments were typically performed to capture 5 cycles (20.5 minutes). Any z-stack data was obtained using a longer chopping period (5 min ON/5 min OFF), with measurement at the end of light or dark segments (images every 0.5 μm for 30 μm total).

A 40X (oil; NA 1.30) objective lens was used for all pH experiments, with 2X zoom to reduce variations in laser intensity across the field of view. A resolution of 1024 x 1024 pixels was used with a scan speed of 600 Hz. Laser intensities were minimised along with sampling frequency (10 s) and dwell time to minimise photodamage. Excitation/emission settings for the various channels are given in Table S1. Time-lapse videos and z-stacks were saved as TIFF stacks for processing in ImageJ.

Table S1. Confocal microscope excitation-emission settings used for pH (BCECF) experiments.

Channel	Excitation wavelength (Laser intensity)	Emission band (Gain)
Autofluorescence	660 nm (2.5 %)	700-750 nm (10)
BCECF (Peak)	488 nm (2.5 %)	525-555 nm (10)
BCECF (Isosbestic point)	448 nm (2.5 %)	525-555 nm (10)

Ratiometric Quantification of pH with BCECF:

Fluorescence images taken of samples with BCECF were background subtracted prior to quantification, to remove the space occupied by cells. This was done using a mask created from the BCECF Peak channel by applying a customised ImageJ Macro:

- run("Convert to Mask", "background=Dark calculate black");
- run("32-bit");
- setThreshold(1.0000, 1e30)
- run("NaN Background", "stack");
- run("Divide...", "value=255.000 stack");

The Peak and Isosbestic point channels were then multiplied by the mask as 32 bit images and quantified across the time axis. Microsoft Excel was used for averaging, division (Peak/Isos) and conversion to pH values using the linear equation generated from the calibration curve. Graphs were made in OriginLab.

The calibration curve was constructed by adjusting solutions of BG11 media to various pHs in the range of 5.5-9.0. One by one, these solutions were added to a microscopy plate on the confocal microscope along with BCECF at 5 μM. Images were recorded (setting as in Table S1)

and processed the same as the experimental data. Linear regression was performed in OriginLab.

Operando Membrane Potential Measurements with ThT

For chopped-light membrane potential experiments, light chopping was performed on a cycle of 1 min ON/1.5 min OFF starting 10 seconds after the time-lapse was initiated. Experiments were typically performed to capture 8 cycles (20 minutes). Any z-stack data was obtained using a longer chopping period (5 min ON/5 min OFF) with measurement at the end of light or dark segments (images every 0.5 µm for 30 µm total).

A lower magnification objective lens (20X air; NA 0.75) was used to capture a greater number of cells within the field of view. A slower scan speed (200 Hz) was used to adapt to this, and the channels were set as shown in Table S2. Time-lapse videos and z-stacks were saved as TIFF stacks for processing in ImageJ and with custom python scripts.

Table S2. Confocal microscope excitation-emission settings used for membrane potential (ThT) experiments.

Channel	Excitation wavelength (Laser intensity)	Emission band (Gain)
Autofluorescence	660 nm (1 %)	700-750 nm (20)
ThT	448 nm (1 %)	470-510 nm (20)

Quantification of Membrane Potential:

ThT fluorescence intensity was quantified from confocal microscopy images using a custom python script. In brief, for each frame, a mask was created from the autofluorescence channel, by applying a gaussian blur, setting a threshold using Otsu's method (skimage.filters.threshold_otsu), then selecting all pixels in the blurred autofluorescence image that were above that threshold. This mask was then applied to the ThT channel to obtain intracellular fluorescence.

ThT fluorescence was used as a proxy for V_m using Equation 2, with control parameters to account for background fluorescence, adapted from Ehrenber *et al.* (1998) and de Souza-Guerreiro *et al.* (2023).[4,5]

$$\Delta V_m = \frac{(p_{xi} - I_{auto}) - R_{Dex}(I_{bkg} - I_{auto,\,bkg})}{(p_{x0} - I_{auto}) - R_{Dex}(I_{bkg} - I_{auto,\,bkg})}$$

Equation 2. Modified Nernst equation for calculation of ΔV_m from ThT fluorescence intensity.

Where:

- p_{xi} is the mean ThT pixel intensity for frame of interest,
- p_{x0} is the mean ThT pixel intensity in the first frame,
- I_{auto} is the autofluorescence of cells in the ThT channel, with no ThT added,
- I_{bkg} is the mean background intensity, with ThT added,
- $I_{auto,\,bkg}$ is the mean background intensity in the ThT channel, with no ThT added,
- R_{Dex} is the proportion of out-of-focus fluorescence collected under imaging conditions. Calculated by adding the cell-impermeable dye Fluorescein Isothiocyanate-Dextran (FITC-Dextran, 5 mg/mL) and measuring the ratio between fluorescence outside and inside of cells (internal fluorescence here coming from out-of-focus light).

This equation assumes that extracellular ThT fluorescence is negligible and can be ignored, and uses the change in intracellular ThT fluorescence from the start condition.

Fluorimetry

Fluorescence spectra were taken with an Agilent Cary Eclipse fluorimeter using an excitation wavelength of 660 nm for *Synechocystis* cells (7.5 $nmol_{Chla}$/mL), and 448 nm and 488 nm for BCECF (5 or 10 μM). A slit width of 2.5 nm was used for all BCECF samples while 5 nm was used for all other samples. A solvent blank was zeroed before each reading.

Oxygen Evolution

Oxygen evolution experiments were conducted using a Pyroscience OXROB10-HS sensor probe, using cell suspensions (2 mL) at a density of 25 $nmol_{Chla}$/mL. Saturating red light (680 nm, 1500 μmol photons/m^2s) was used to initiate photosynthesis, under constant stirring.

Statistics

All results are presented as the mean +/- standard deviation of 3 biological replicates unless stated otherwise. Where pair-wise comparisons were made, a two-tailed student's t-test was used, using a paired approach when the measurements were dependent.

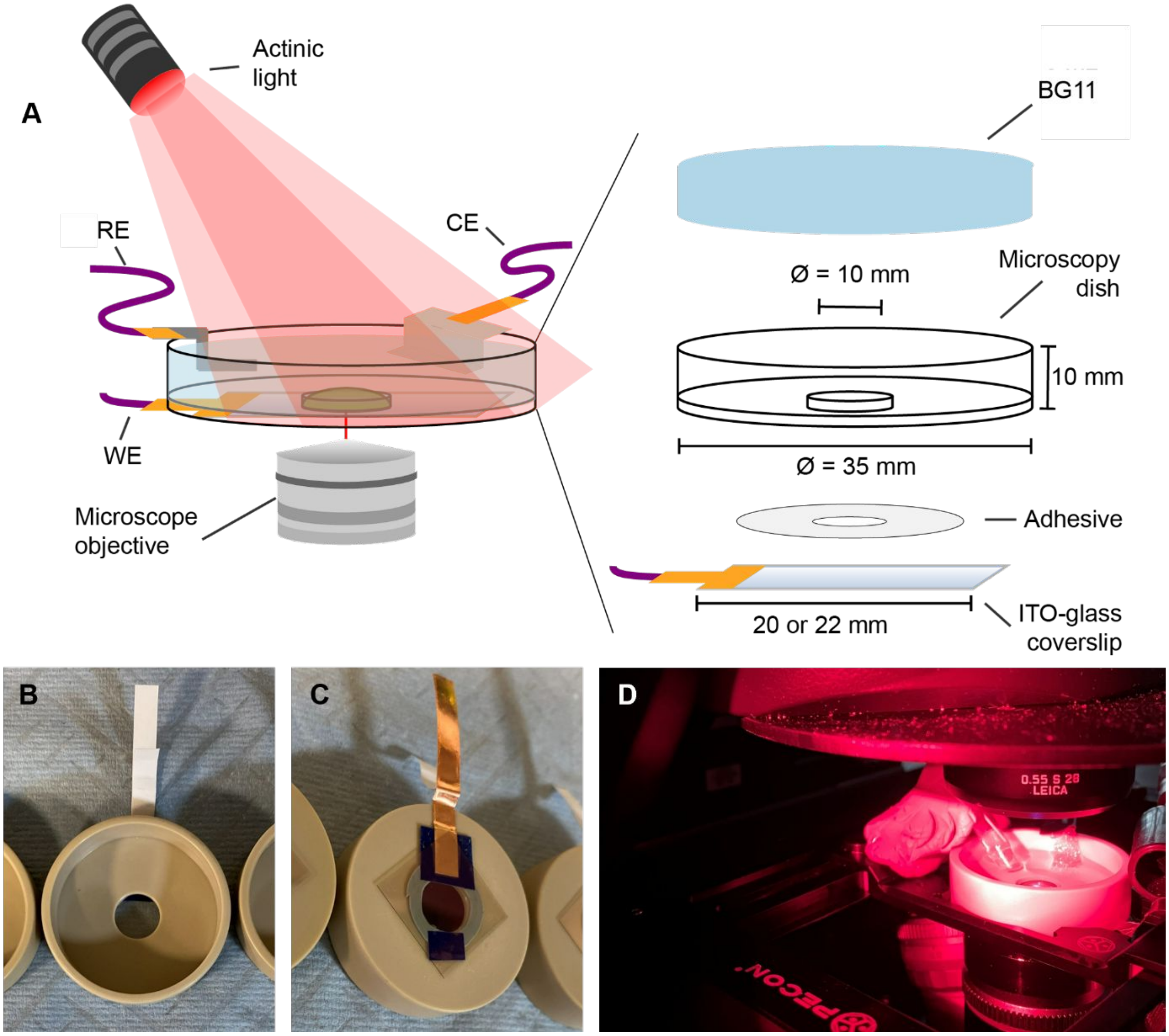

Figure S1. Assembly and set-up of the electrochemistry-adapted microscopy plates used for *operando* investigation of the *Synechocystis*-electrode interface. **(A)** *Operando* microscopy set-up showing positioning of the working (WE), counter (CE), and reference (RE) electrodes and the actinic 680 nm LED light. Expansion of microscopy plate shows the dimensions of each layer from the indium tin oxide (ITO) coverslip to the BG11 electrolyte medium. **(B)** Photograph of the top view and bottom view **(C)** of the ITO coverslip WE installed on the microscopy plate. **(D)** Photograph of the microscopy plate set-up on the confocal microscope with the Pt mesh CE and Ag/AgCl RE suspended in solution and the LED directed down onto the plate.

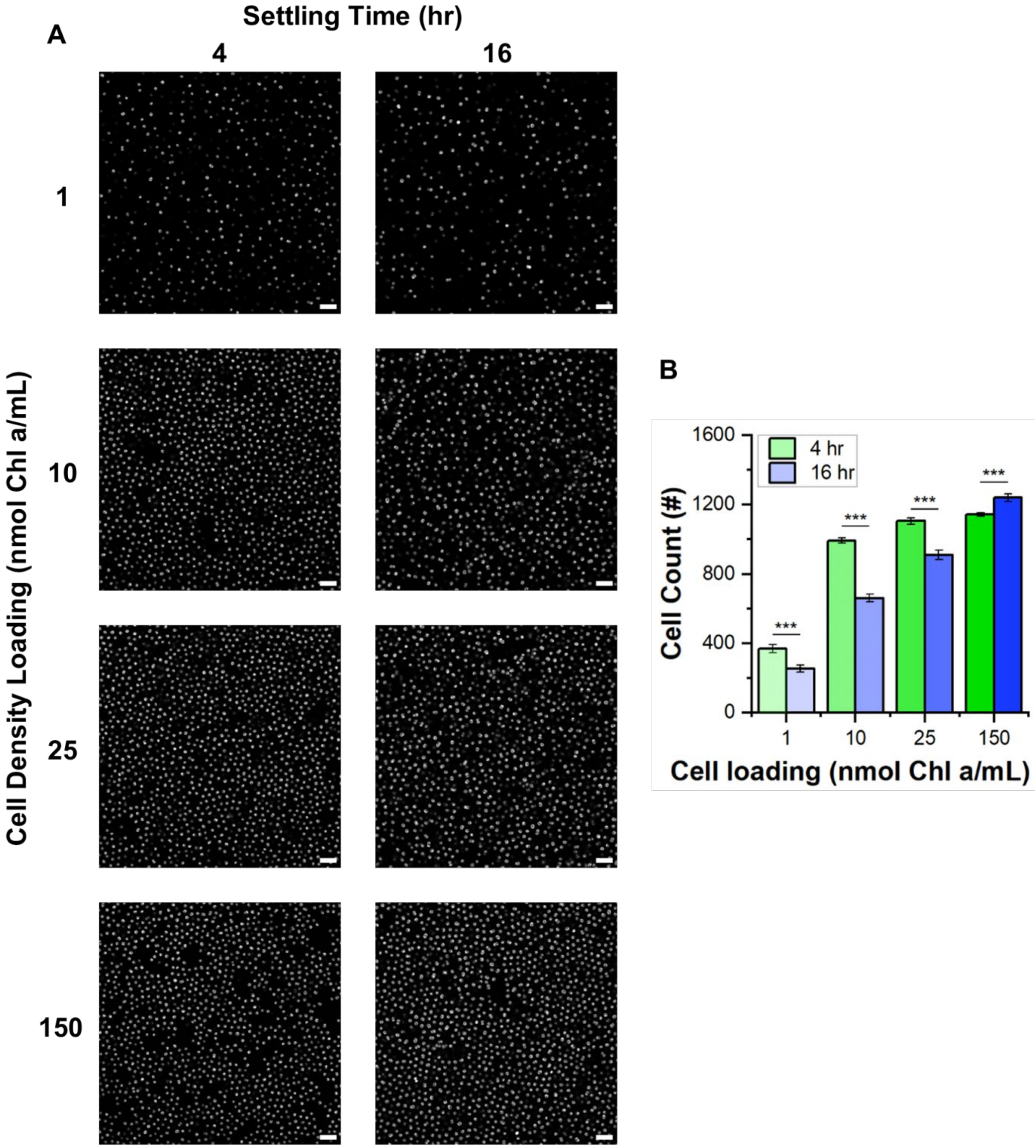

Figure S2. Quantification of the cell layer formed on the coverslip electrode after 4 or 16 hours of settling time. *Synechocystis* cells grown to an OD_{750} = 1.0 +/- 0.1 were concentrated or diluted into fresh BG11 media (pH 7.5) to 1, 10, 25, or 150 $nmol_{Chla}$/mL prior to loading 200 uL onto the electrochemistry-adapted microscopy plates. **A** Confocal fluorescence images showing *Synechocystis* cells via pigment autofluorescence (excitation 660 nm, emission 700-750 nm). Images are representative of 3 replicates. Scale bar = 10 µm. **B** Cell count quantified from the images represented in A, presented as the mean and standard deviation (N = 3 biological replicates).

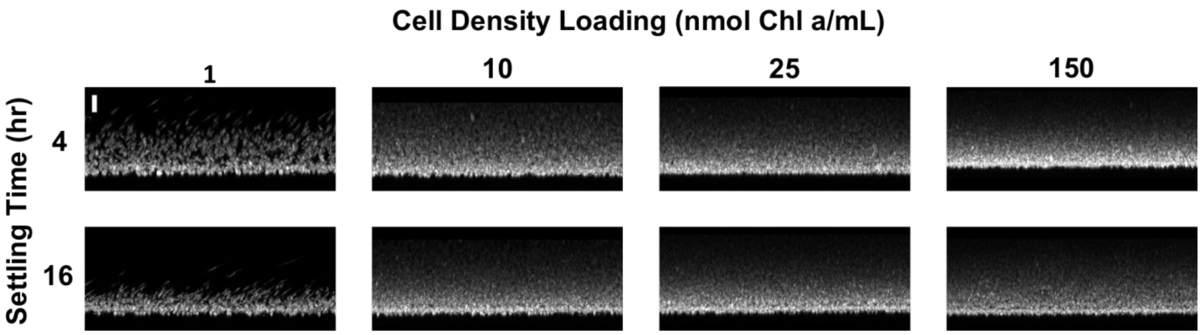

Figure S3. *Synechocystis* cell layers observed along the Z-axis on a confocal microscope (excitation 660 nm, emission 700-750 nm). 3D reconstruction performed in ImageJ. Images are representative of three biological replicates. Scale bar = 10 um.

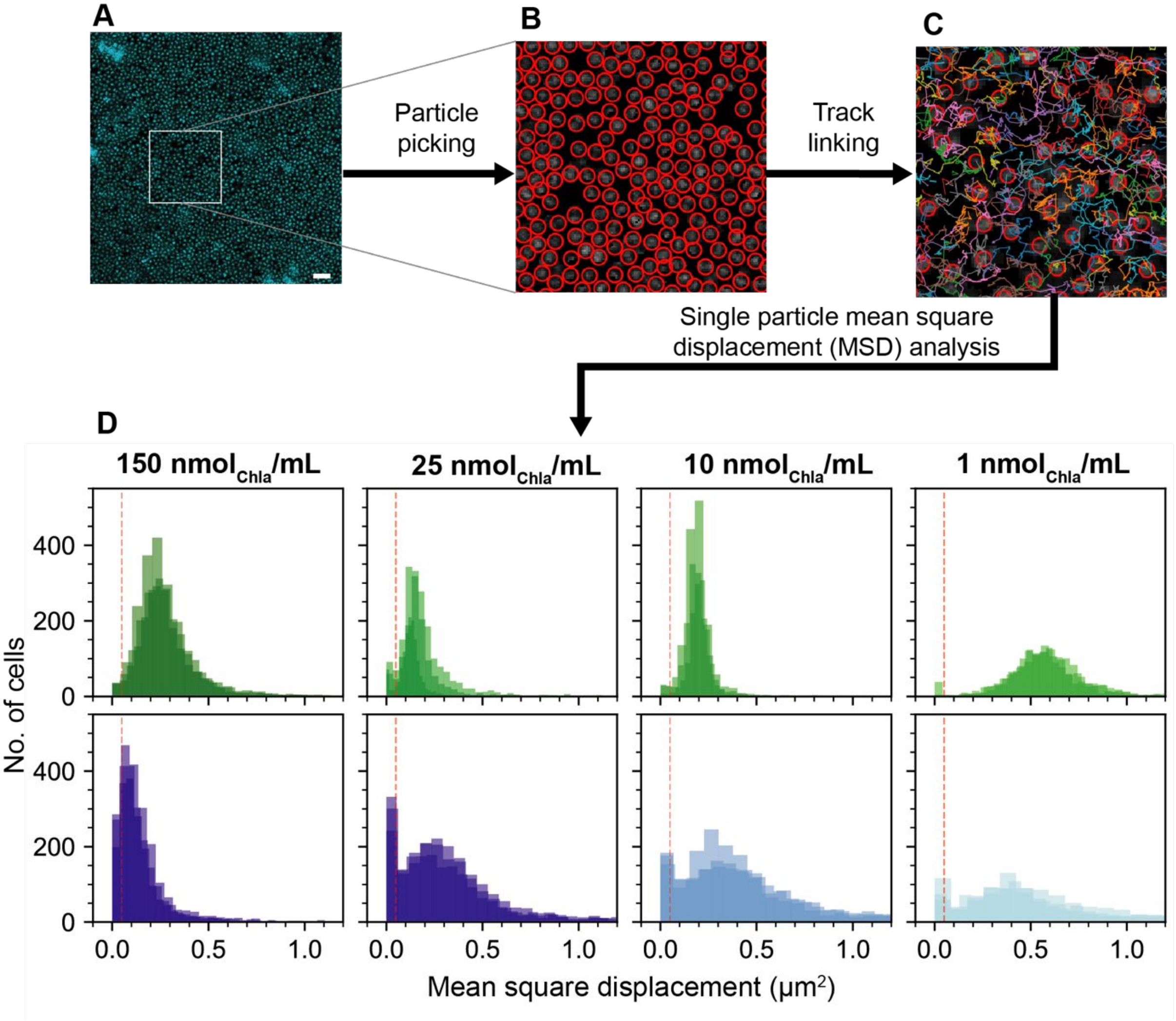

Figure S4. Workflow for mobility analysis presented in Figure 2C and 5A. For each condition, a 5 minute time-lapse of the cells at the electrode surface was collected (images taken every 10 seconds). **A** Representative image from the time-lapse **B** Particles were picked using a custom python code employing the trackpy particle tracking algorithm. **C** These picked particles were then linked into tracks, and their Mean Square Displacement calculated **D** Histograms of mean square displacements (MSD) of populations of *Synechocystis* cells at different loading times and densities, calculated from linked tracks as described in Fig S4. For each condition three histograms are shown overlayed (N = 3 biological replicates), and the threshold of 0.05 μm^2 used in **Figure 2Cii** shown as a red dotted line.

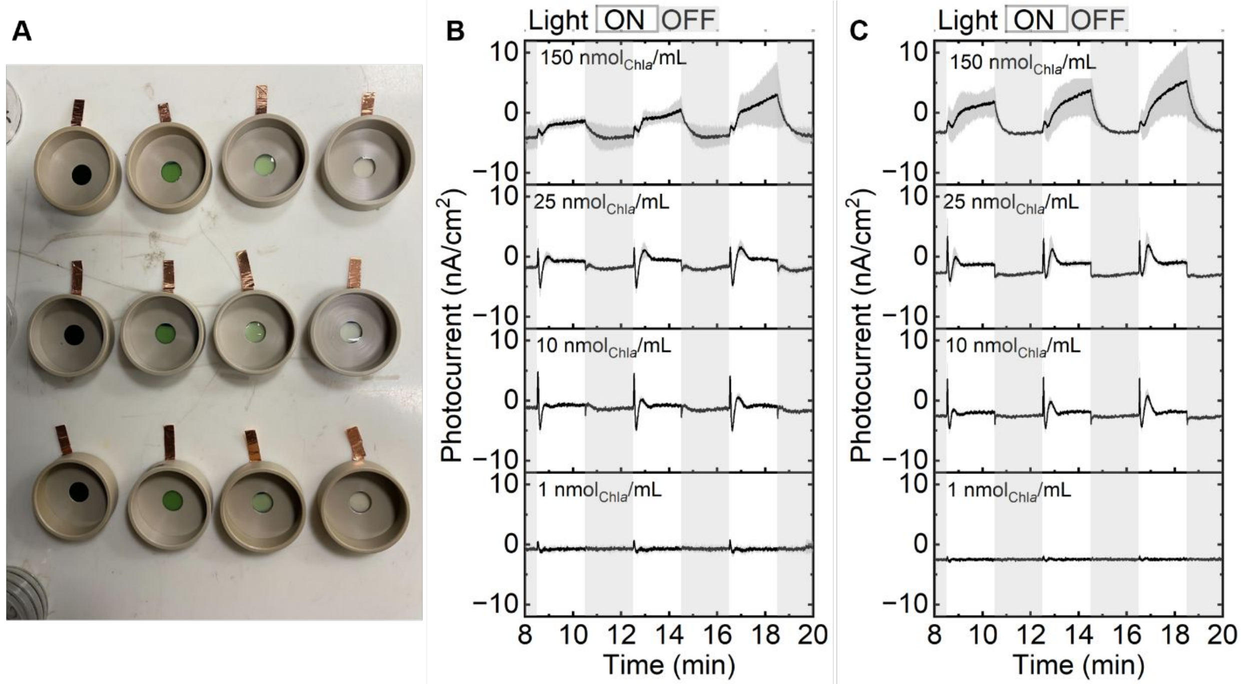

Figure S5. Photoelectrochemistry of *Synechocystis*-electrode biohybrids. **A** Photograph of the cells prepared for settling at different concentrations (150, 25, 10, or 1 $nmol_{Chla}$/mL). Cells were settled for 4 (**B**) or 16 (**C**) hours in the dark prior to measurement, with data plotted after the curves had become consistent (cycles 3-5). Chronoamperometry mode was used (E = 0.1 V vs. Ag/AgCl) with a three-electrode configuration (ITO working electrode, platinum mesh counter electrode, Ag/AgCl reference electrode). Light chopping was performed with a 680 nm LED (150μmol photons/m^2s) on a cycle of 2 min ON/2 min OFF (N = 3 biological replicates).

Table S3. Comparison of Fluorescent pH Indicators.Color coding reflects the *a priori* compatibility expected when used with *Synechocystis* displaying autofluorescence (green = good, yellow = okay, red = poor compatibility).

**pH Indicator**	Intracellular or extracellular	Ratiometric/ quantitative?	pKa	Dual excitation or dual emission	Excitation / emission maxima (nm)	Quantum yield (see Refs for conditions)	Previous use with cyanobacteria
BCECF [Ref. [6]]	Extracellular	Yes	7.0	Excitation	503/525 (isosbestic point: excitation 440 nm)	0.84	-
BCECF-AM [Ref. [6,7]]	Intracellular	Yes	7.0	Excitation	503/525 (isosbestic point: excitation 440 nm)	0.84	*S. elongatus*
C.SNARF-1 [Ref. [6]]	Extracellular	Yes	7.5	Emission	544/575 (phenolic), 583/631 (phenolate)	0.03 (phenolic), 0.09 (phenolate)	-
C.SNARF-1-AM [Ref. [6]]	Intracellular	Yes	7.5	Emission	544/575 (phenolic), 583/631 (phenolate)	0.03 (phenolic), 0.09 (phenolate)	-
Acridine orange [Ref. [8,9]]	Intracellular	No	10.5	Emission	492/530 (monomer), 465/655 (dimer)	0.46	*Synechocystis, S. elongatus*

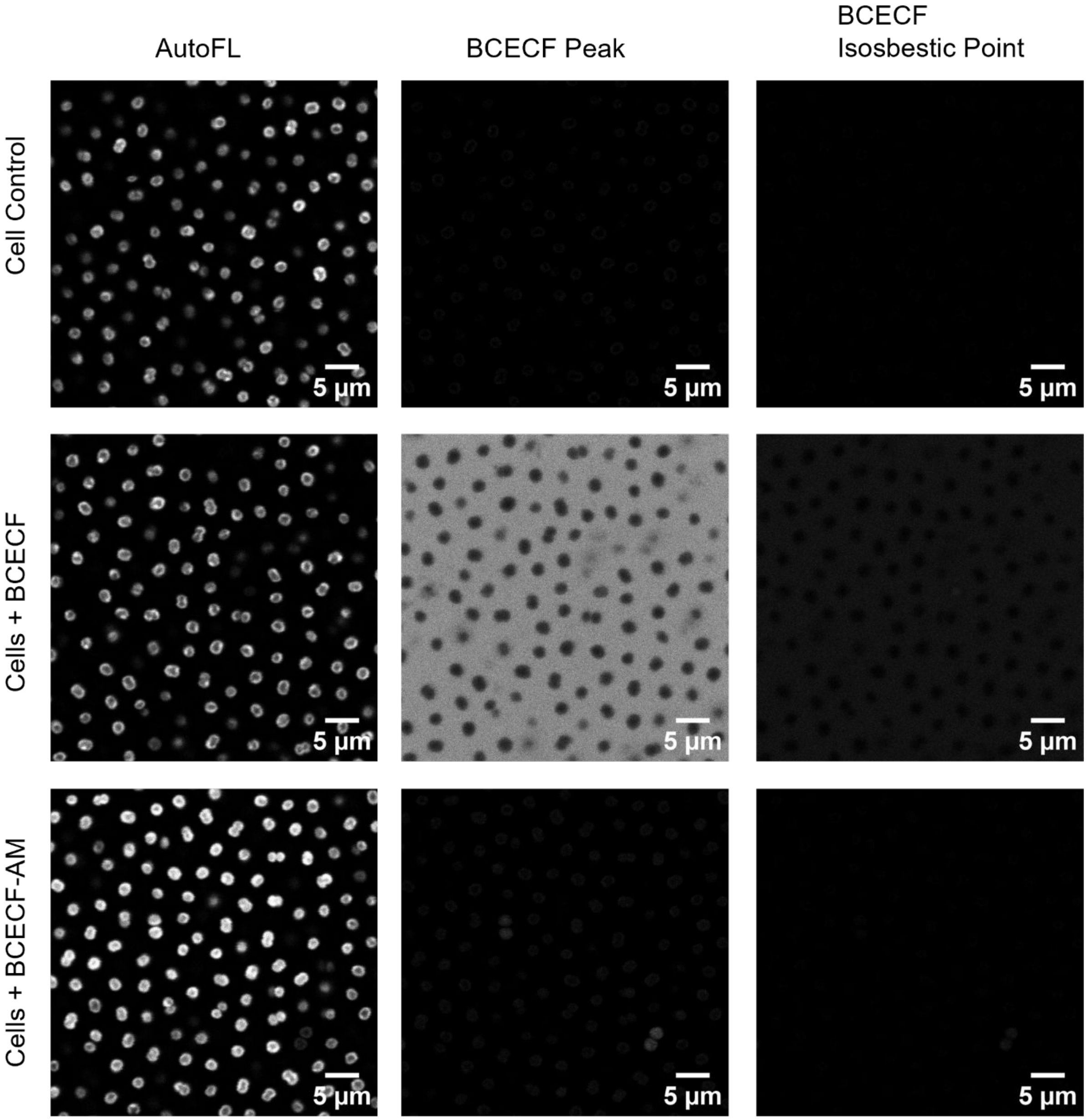

Figure S6. Screening of fluorescent pH probes- BCECF and BCECF-AM. Confocal fluorescence images showing the fluorescence output of samples containing *Synechocystis* cells with or without BCECF or BCECF-AM (5 μM) in BG11 (pH 7.5). The imaging channels were set as follows: cell autofluorescence (AutoFL; excitation 660 nm, emission 700-750 nm); BCECF peak fluorescence (excitation 488 nm, emission 525-555 nm); BCECF isosbestic point (excitation 448 nm, emission 525-555 nm). As the acetoxymethyl groups of BCECF-AM are cleaved by intracellular esterases to activate it, the spectroscopic properties are the same as BCECF. In the case of BCECF-AM, cells were incubated for 25 minutes prior to imaging.

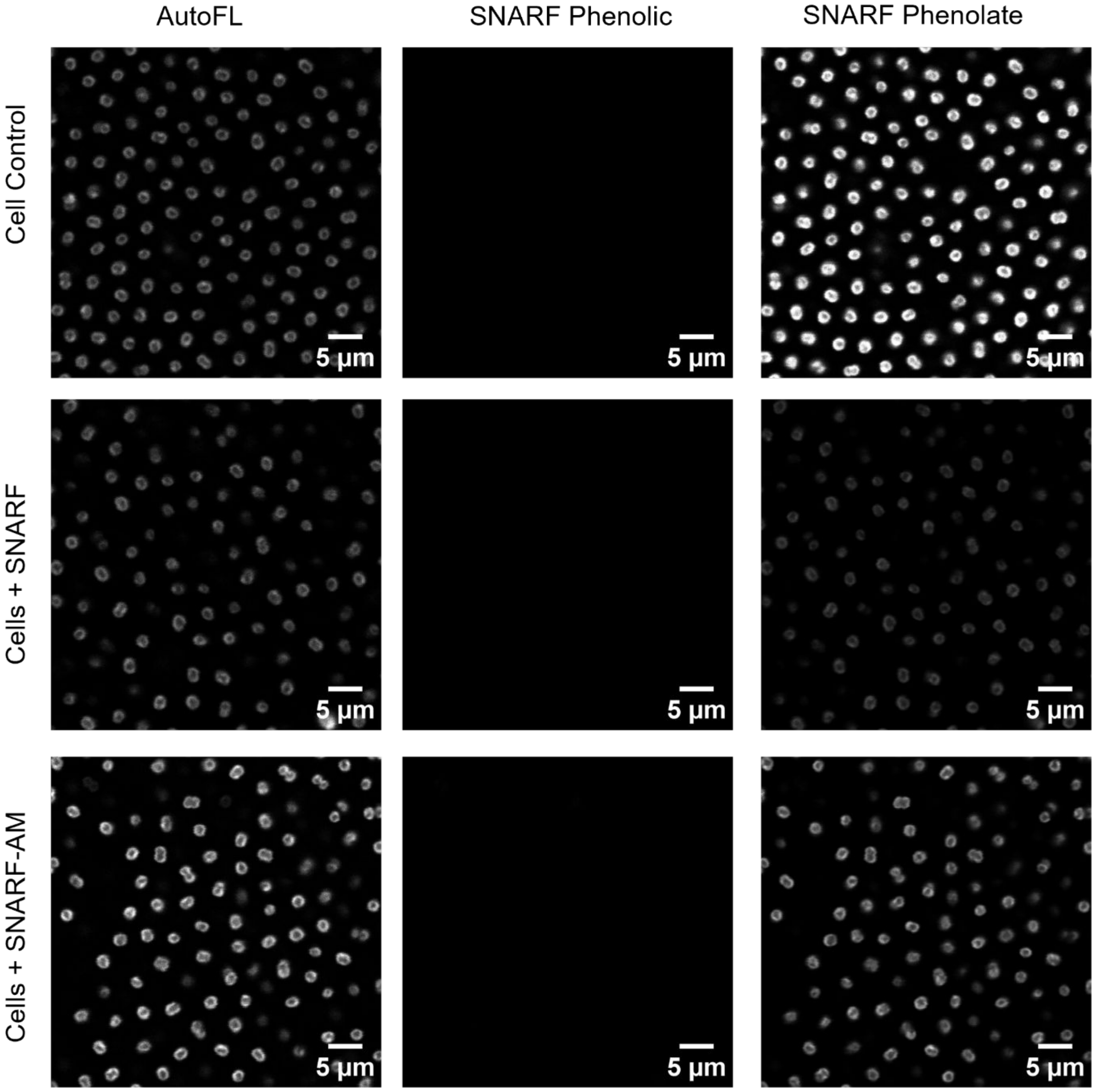

Figure S7. Screening of fluorescent pH probes- C.SNARF-1 (SNARF) and C.SNARF-1-AM (SNARF-AM). Confocal fluorescence images showing the fluorescence output of samples containing *Synechocystis* cells with or without SNARF or SNARF-AM (5 μM) in BG11 (pH 7.5). The imaging channels were set as follows: cell autofluorescence (AutoFL; excitation 660 nm, emission 700-750 nm); SNARF (phenolic) fluorescence (excitation 540 nm, emission 550-580 nm); SNARF (phenolate) fluorescence (excitation 590 nm, emission 610-640 nm). As the acetoxymethyl groups of SNARF-AM are cleaved by intracellular esterases to activate it, the spectroscopic properties are the same as SNARF. In the case of SNARF-AM, cells were incubated for 25 minutes prior to imaging.

Note: The SNARF (phenolate) channel overlapped substantially with cell autofluorescence, resulting in an inability to distinguish the real signal.

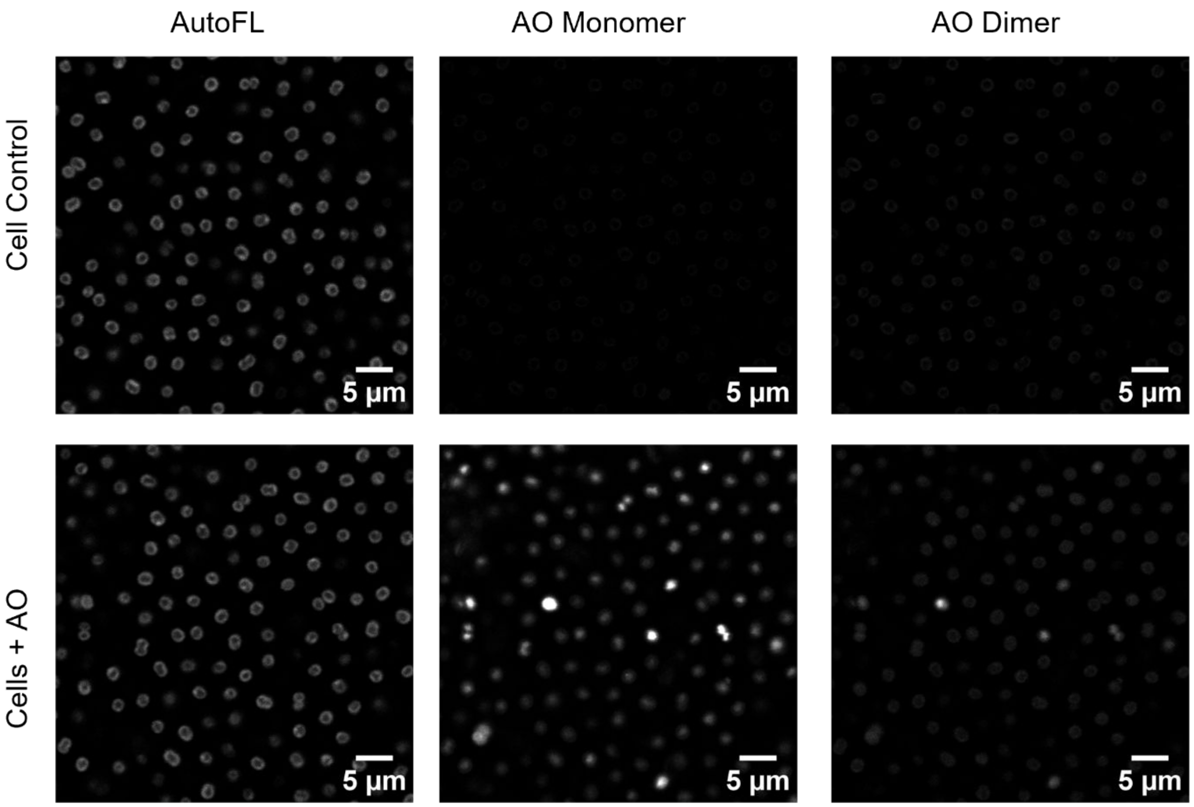

Figure S8. Screening of fluorescent pH probes- acridine orange (AO). Confocal fluorescence images showing the fluorescence output of samples containing *Synechocystis* cells with or without AO (5 µM) in BG11 (pH 7.5). The imaging channels were set as follows: cell autofluorescence (AutoFL; excitation 660 nm, emission 700-750 nm); AO monomer (excitation 488 nm, emission 515-535 nm); AO dimer (excitation 488 nm, emission 600-620 nm). Cells were incubated with the probe for 25 minutes before imaging.

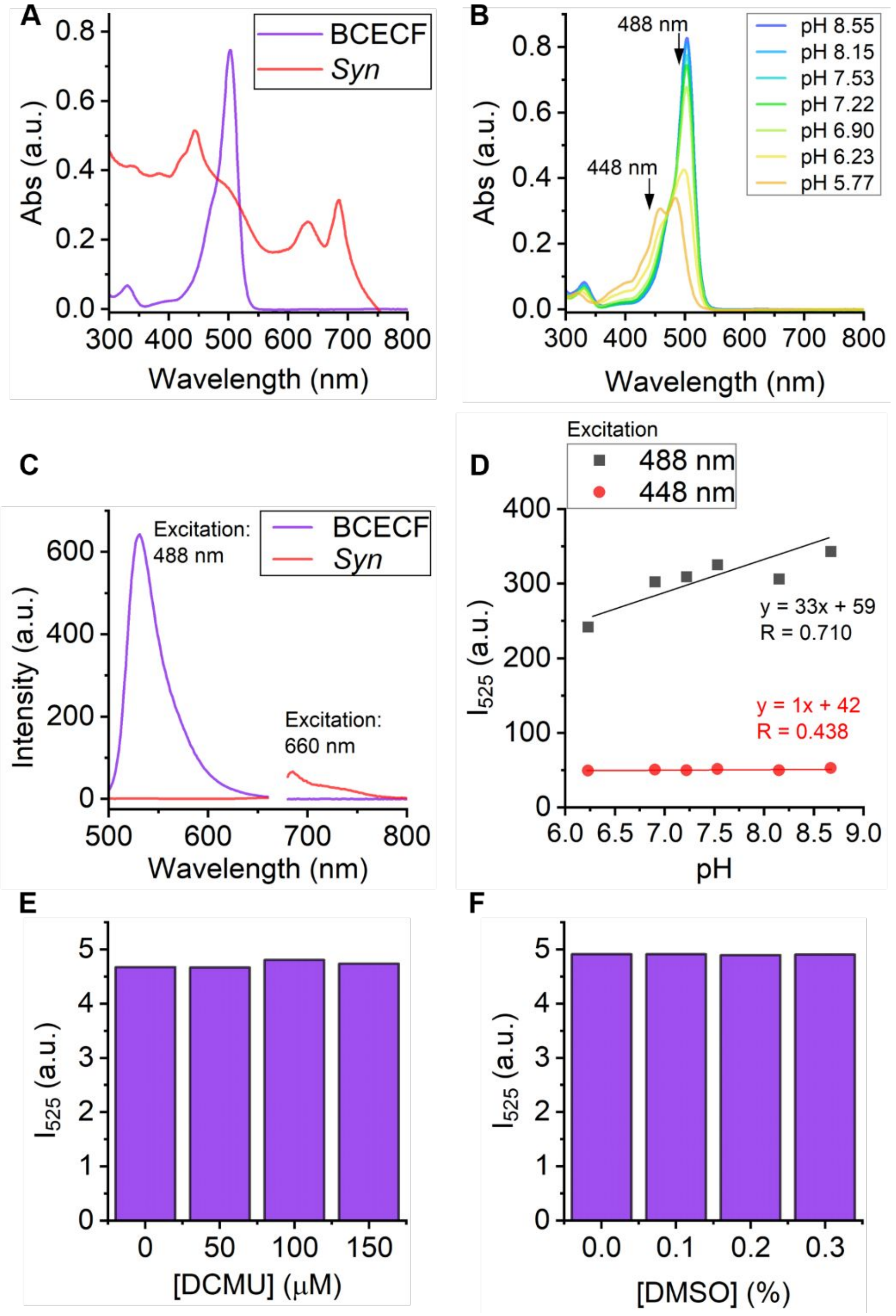

Figure S9. Spectroscopy demonstrating BCECF as a useful pH indicator in BG11 medium. **A** Absorption spectra of BCECF and *Synechocystis* cells (10 $nmol_{Chl a}$/mL) after background subtraction. **B** pH titration series of BCECF showing the pH-dependent absorption at 488 nm and the non-pH-dependent absorption at 448 nm. **C** Fluorescence intensity of BCECF solutions (10 μM) and *Synechocystis* solutions (7.5 $nmol_{Chl a}$/mL) in BG11 medium. Excitation wavelengths of 448 or 660 nm were applied. **D** Fluorescence intensity at 525 nm during pH titration of BCECF when excitation at 488 or 448 nm was used. **E** Fluorimetry of BCECF titrated with DCMU or **F** DMSO with the BCECF ratio at 525 nm plotted. All tests used BCECF at 5 μM in BG-11 (pH 7.5) unless otherwise noted (N = 1).

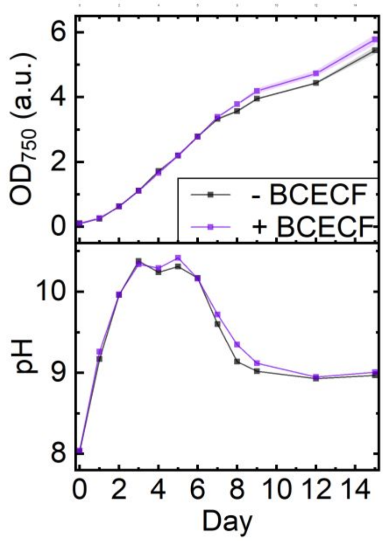

Figure S10. Growth curves of *Synechocystis* in the presence of BCECF. BCECF (5 μM) was added to freshly inoculated flasks of cells in BG-11 (pH 7.5). The optical density and culture pH were sampled over two weeks from the same aliquots (N = 1).

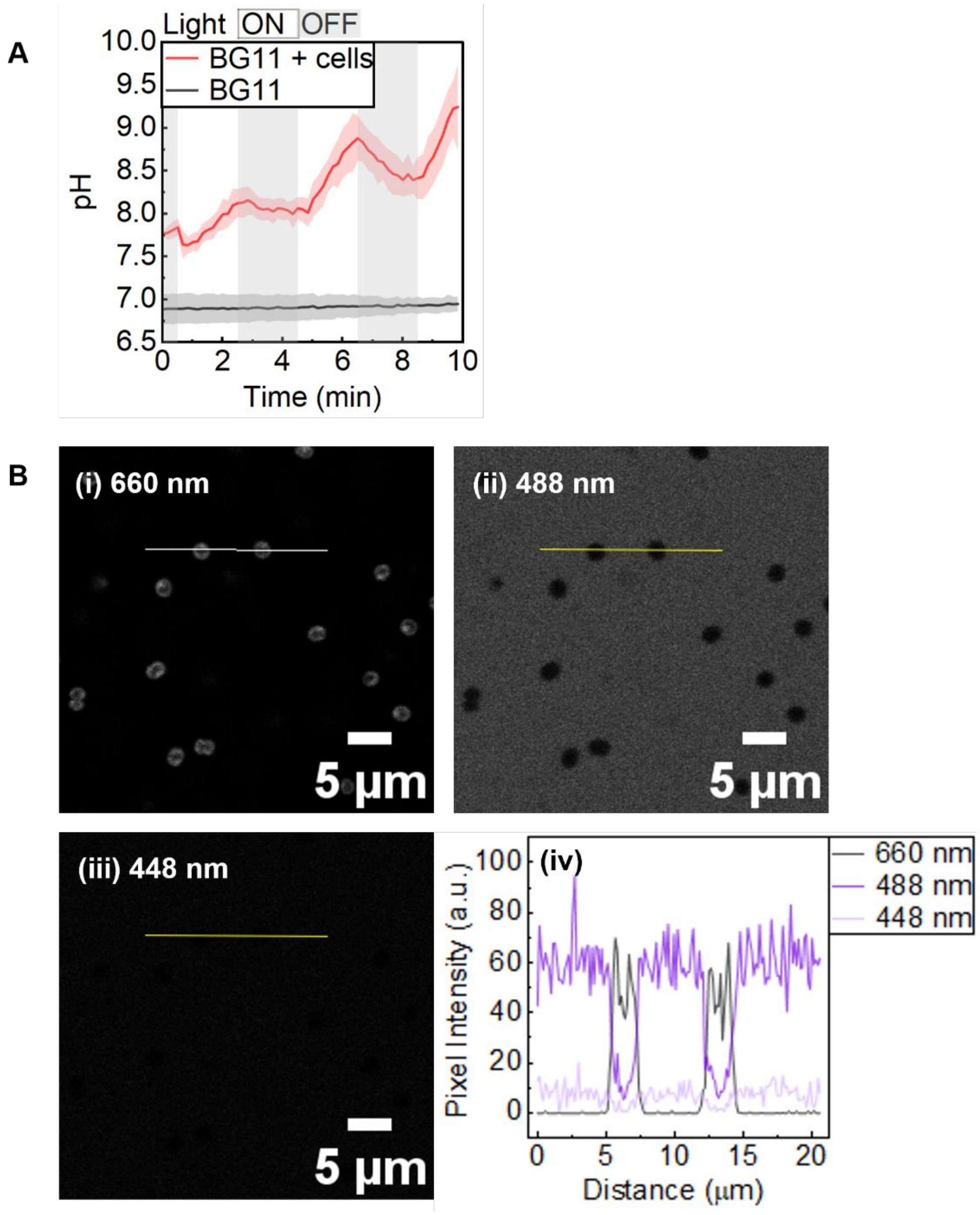

Figure S11. *Operando* pH quantification in the presence or absence of *Synechocystis* cells. **A** Confocal microscopy was performed on a microscopy dish containing BG11 medium containing BCECF (5 µM) which was subject to chopped light (680 nm, 150 µmol photon/m^{2}s). A bare glass coverslip was used instead of an ITO-coated one. Data represent the mean and standard deviation (N = 3, biological replicates where cells were used). **B** Raw fluorescence images for the confocal channels in this work (**i-iii**) with quantification of a linescan across the image stack (**iv**) (N = 1).

Note: intracellular fluorescence, or out-of-focus fluorescence arising from BCECF is negligible, however a mask was used to exclude the intracellular space prior to quantification of BCECF fluorescence nonetheless.

A

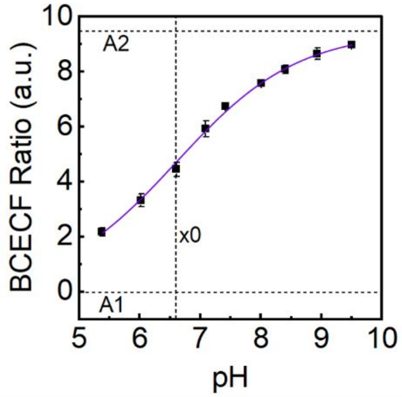

Model	Boltzmann
Equation	y = A2 + (A1-A2)/(1 + exp((x-x0)/dx))
Plot	BCECF Ratio
A1	-0.02221 ± 0.90198
A2	9.47578 ± 0.13287
x0	6.60124 ± 0.21354
dx	1.00211 ± 0.12719
R-Square	0.99898
Adj. R-Sq	0.99837

B

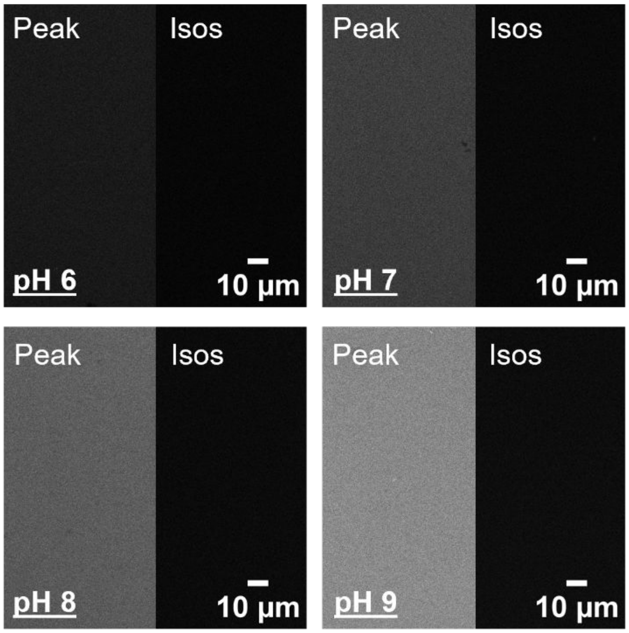

Figure S12. pH calibration curve using BCECF. Confocal microscopy was performed on BG11 solutions at pH values predetermined with a pH meter. **A** Quantification of the BCECF Ratio (peak fluorescence divided by the fluorescence at the isosbestic point – see Methods) and the derived equation for pH calculations. Data represent the mean and standard deviation (N = 3 technical replicates). **B** Examples of confocal images that were used for calibration curve construction. Images are representative of three technical replicates.

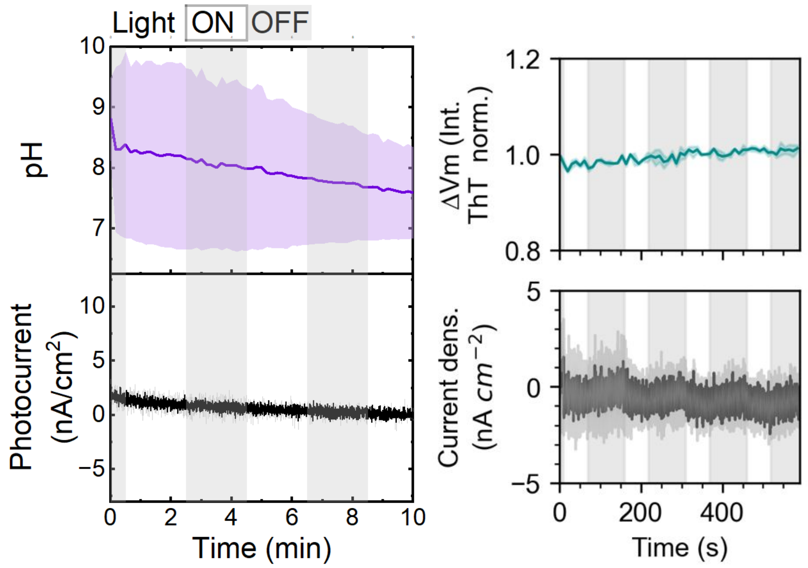

Figure S13. Light-chopping performed on a heat-treated *Synechocsytis* biofilm attached to an indium tin oxide electrode. **Left** pH measured with BCECF (5 uM) in BG11 media, calibrated to quantitative values. **Right** V_m measured with ThT (10 uM). Fluorescent probe data (**top**) and photocurrent density (**bottom**). Cells were heated at 100˚C for 5 mins prior to loading into the microscopy plate for settling. Data represent the mean and standard deviation (N = 2 biological replicates).

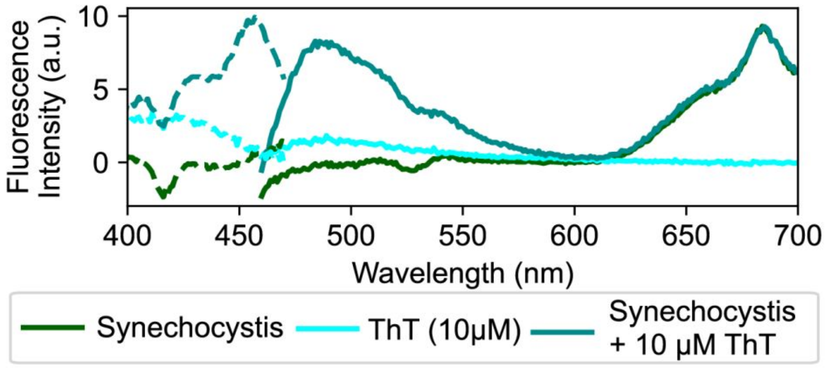

Figure S14. Spectroscopy demonstrating ThT as a useful membrane potential indicator with Synechocystis. Fluorescence excitation (dotted lines) and emission (solid lines) spectra of a liquid *Synechocystis* culture (25 $nmol_{Chla}$/mL; green), ThT (10 μM, cyan), and *Synechocystis* incubated with 10 μM ThT (dark cyan) in BG11 medium (N = 1).

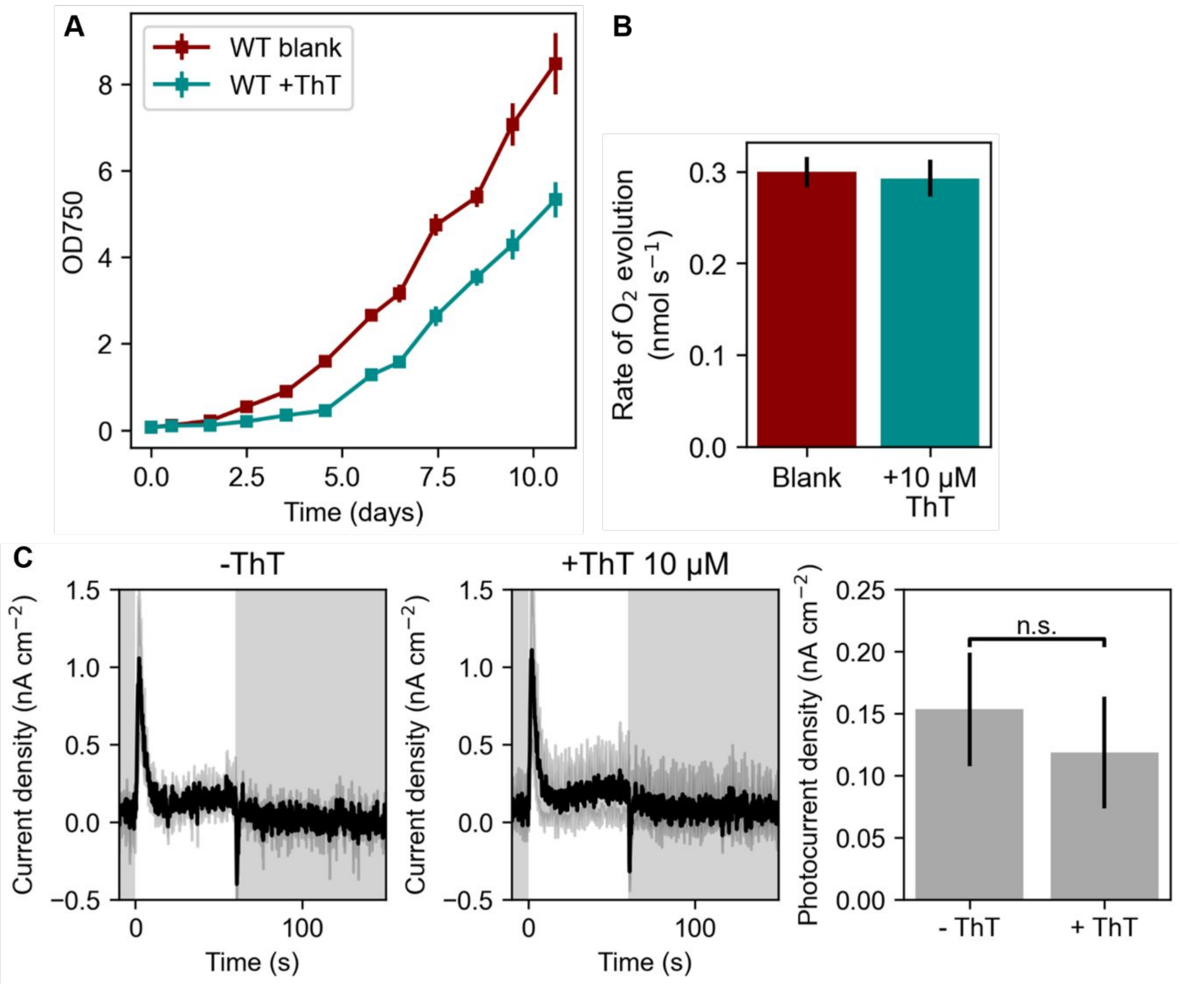

Figure S15. Testing the impact of ThT on *Synechocystis* cell function. **A** Growth curves of *Synechocystis* with or without 10 μM ThT. **B** Short-term oxygen evolution of *Synechocystis* cell cultures (2 mL, 25 nmol$_{Chla}$/mL). **C** Photocurrent measurements of *Synechocystis* cells (25 nmol$_{Chla}$/mL) during chronoamperometry (E = 0.1 V vs. Ag/AgCl) with a three-electrode configuration and light chopping on a cycle of 1 min ON/1.5 min OFF in BG11 media (ITO working electrode, platinum mesh counter electrode, Ag/AgCl reference electrode; 680 nm LED, 150μmol photons/m^{2}s). Data represents the mean and standard deviation (N = 3 biological replicates).

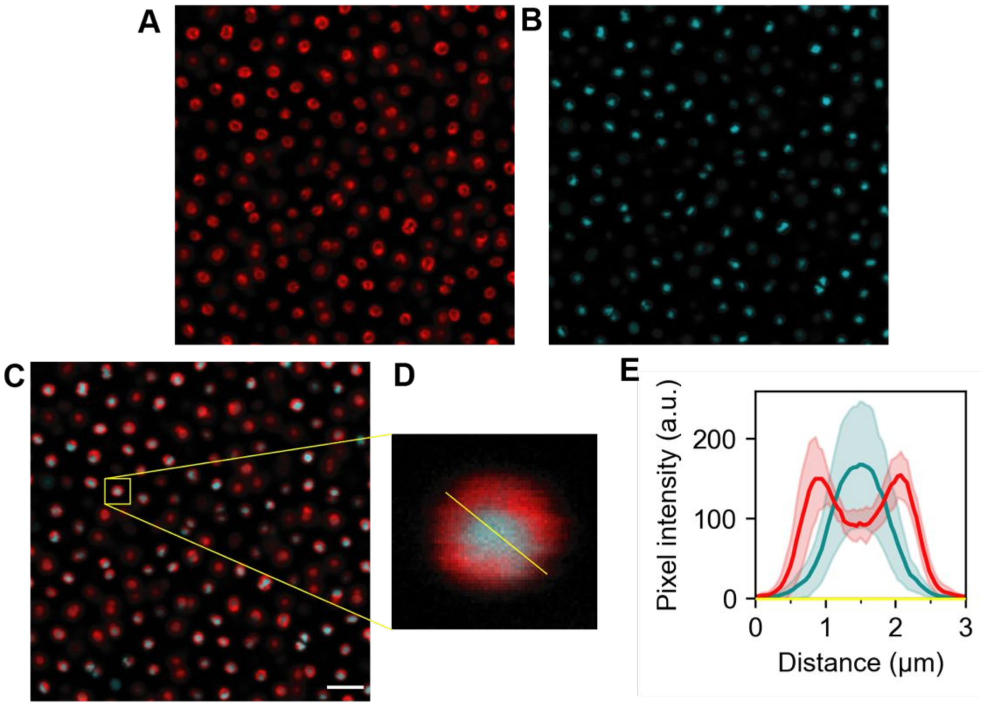

Figure S16. ThT subcellular localisation in *Synechocystis* cells. Representative images of **A** cell autofluorescence (red) **B** ThT fluorescence (cyan), **C** their overlay, and **D** a single cell, showing thylakoid-localised autofluorescence and cytosolic ThT fluorescence. Scale bar: 5 µm. **E** Cross sections of fluorescence intensity across cells, with mean and standard deviation shown for 3 cells in each of 3 biological replicates. Yellow line demonstrative of cross section taken.

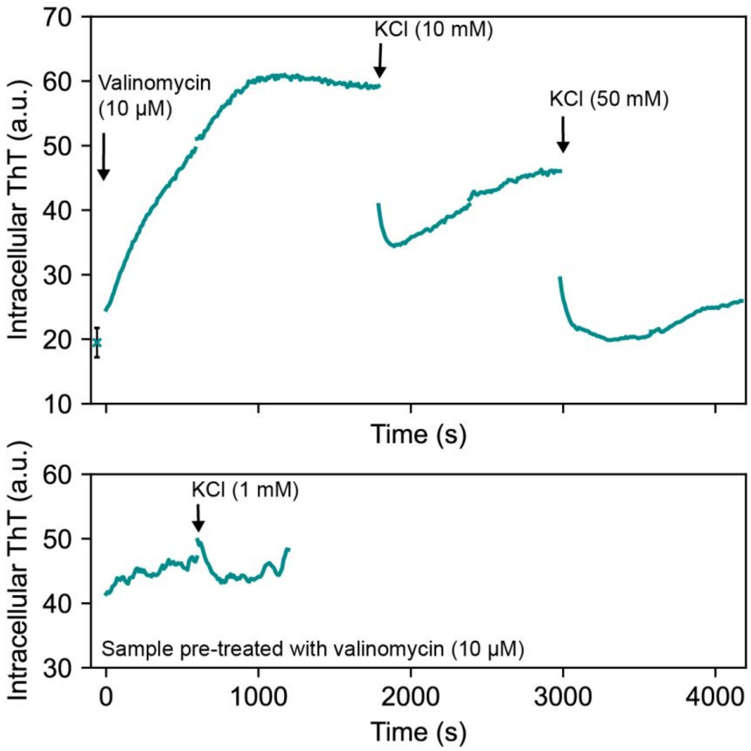

Figure S17. Establishing response of intracellular [ThT] to ionic V_m changes. **Top** 10 µM Valinomycin was added to *Synechocystis* cells (25 $nmol_{Chla}$/mL) incubated with 10 µM ThT, and their intracellular ThT fluorescence tracked by confocal microscopy over the subsequent 30 minutes. After 30 min (t = 1800 s), KCl (10 mM) was added, and after another 20 min (t = 3000 s) KCl (50 mM) was added. **Bottom** in a separate sample, under the same experimental conditions and pre-treated with Valinomycin (10 µM) for 1 hr, intracellular ThT fluorescence was tracked by confocal microscopy, with addition of KCl (1 mM) after 10 min (t = 600). (N = 1)

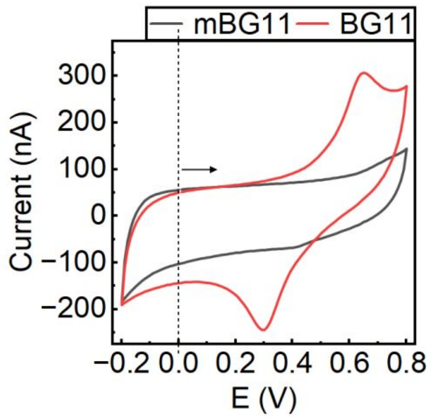

Figure S18. Cyclic voltammetry of BG11 and minimal BG11 (mBG11) media. A three-electrode configuration was used with indium tin oxide working, platinum mesh counter, and Ag/AgCl reference electrodes. Average and standard deviation of 3 consecutive cycles shown.

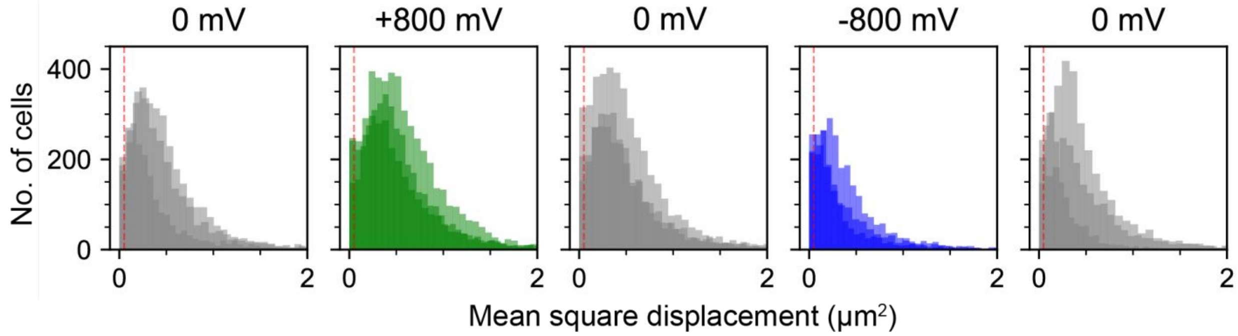

Figure S19. Histograms of mean square displacements (MSD) of populations of *Synechocystis* cells at different applied potentials throughout the potential sweep experiment, calculated from linked tracks as described in Fig S4. For each condition three histograms are shown overlayed (n = 3 biological replicates), and the threshold of 0.05 μm^2 used in **Figure 5A** shown as a red dotted line

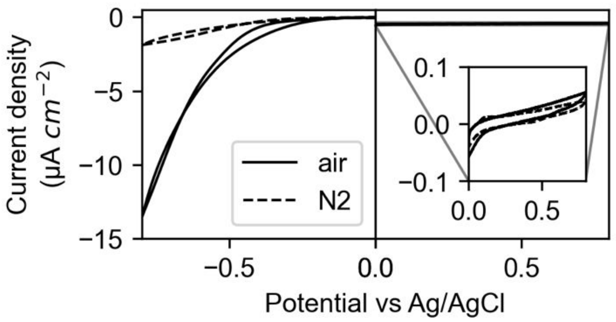

Figure S20. Cyclic voltammetry of minimal BG11 medium before (solid line) and after (dashed line) degassing with N_2 for 30 minutes. Inset shows a zoomed in view of the positive sweep (N = 1).

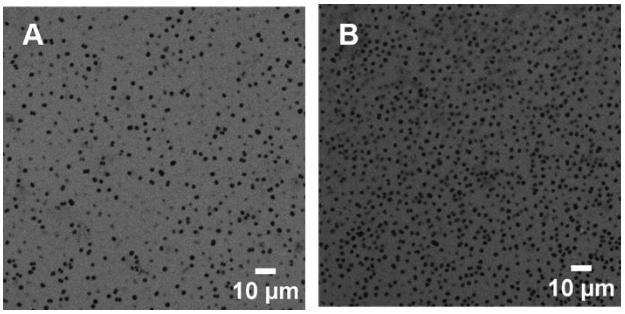

Figure S21. Confocal imaging of BCECF around *Synechocystis* cells during a negative potential sweep from **A** 0.0 V to **B** -0.8 V vs. Ag/AgCl. A three-electrode configuration was used with indium tin oxide working, platinum mesh counter, and Ag/AgCl reference electrodes (N = 1).

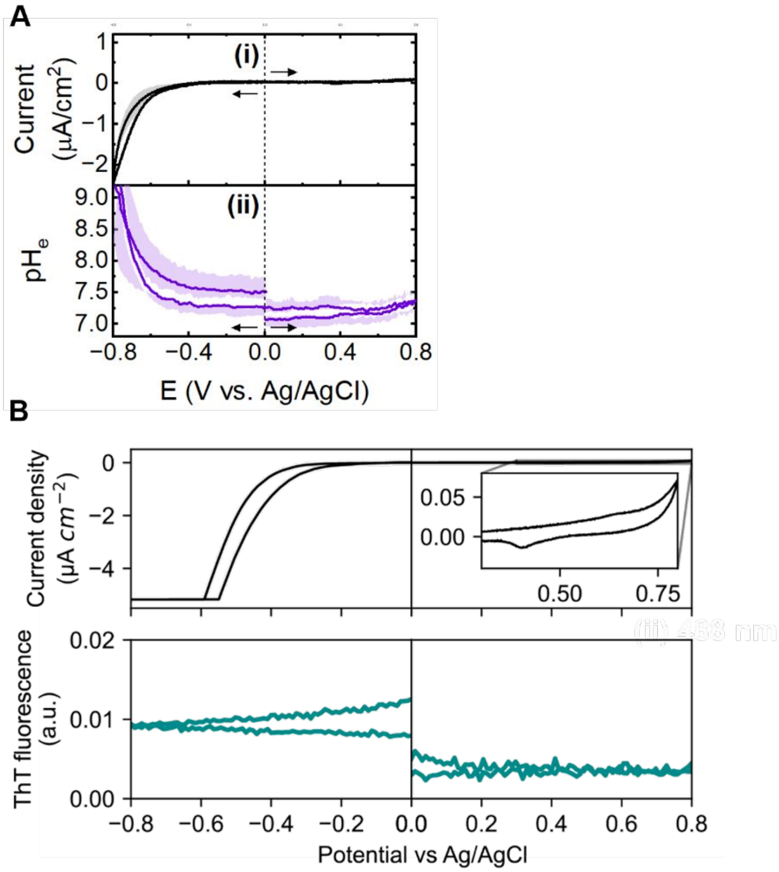

Figure S22. Abiotic controls for *operando* extracellular pH (**A** pH_e) and change in membrane potential (**B** ΔV_m) analysis during cyclic voltammetry using the fluorescent probes BCECF and ThT. Scan rate 1 mV/s. A three-electrode configuration was used (indium tin oxide working, platinum mesh counter, Ag/AgCl reference electrodes, in minimal BG11 media. Data presented as the mean and standard deviation (N = 3 technical replicates for A; N = 1 for B).

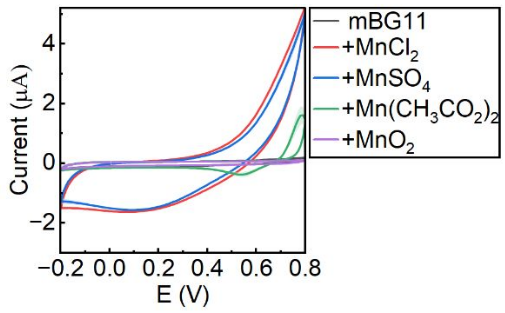

Figure S23. Cyclic voltammetry of various manganese complexes (14 uM) in mBG11 media (pH 7.5). A three-electrode set-up was used: indium tin oxide-coated glass working electrode, platinum mesh counter electrode, Ag/AgCl reference electrode. The scan was started at 0 V and scanned anodic first. The data represent the mean and standard deviation of three consecutive cycles.

Supplementary Note 1. Sensitivity measurements of ThT

To determine the sensitivity and limit of detection of ThT, we carried out a series of control experiments employing valinomycin, an antibiotic which selectively permeabilises the cell membrane to K^+ ions, and a KCl titration.

First, valinomycin was added to a biofilm of *Synechocystis* cells incubated with ThT, resulting in a large increase in membrane potential, as K^+ ions left the cell. Intracellular [K^+] we estimated at ~10 mM,[10] significantly higher than extracellular [K^+] (350 μM in BG11). This efflux corresponds to:

$$\Delta V_m = -\frac{RT}{zF} ln(\frac{0.000350}{0.010}) = -84.6\, mV$$

This equation gives an upper limit to the range of detectable ΔV_m and confirms that ThT is able to respond to changes in membrane potential induced by ionic effects.

Next, we carried out KCl titration experiments, to determine the sensitivity of ThT as a membrane potential probe in *Synechocystis*. When *Synechocystis* is treated with valinomycin, K^+ should equilibrate across the membrane, and any excess Cl^- ions should accumulate outside the cell, thus altering V_m. If we assume that $[K^+]_{int}$:$[K^+]_{ext}$ remains stable in the presence of valinomycin, then we can estimate ΔV_m at two timepoints (t0 and t1) based on extracellular [Cl^-].

$$V_m^{t1} - V_m^{t0} = \frac{RT}{zF}(ln(\frac{[\mathrm{Cl}-]_{outside}^{t1}}{[\mathrm{Cl}-]_{inside}^{t1}}) - ln(\frac{[\mathrm{Cl}-]_{outside}^{t0}}{[\mathrm{Cl}-]_{inside}^{t0}}))$$

simplifies to:

$$V_m^{t1} - V_m^{t0} = \frac{RT}{zF} ln(\frac{[\mathrm{Cl}-]_{outside}^{t1}}{[\mathrm{Cl}-]_{outside}^{t0}})$$

if we assume intracellular [Cl^-] remains effectively constant. This can be expanded to:

$$\Delta V_m = \frac{RT}{zF} ln(\frac{[\mathrm{Cl}-]_{outside}^{t0} + [\mathrm{Cl}-]^{added}}{[\mathrm{Cl}-]_{outside}^{t0}})$$

BG11 contains 324 μM $CaCl_2$, so [Cl^-] = 0.000624. With this we get a relationship between ΔV_m and [Cl^-]:

$$\Delta V_m = \frac{RT}{zF} ln(1 + \frac{[Cl-]_{added}}{0.000624})$$

However, because [K^+] distributes evenly across the membrane in the presence of valinomycin, here $[Cl^-]_{added}$ is not the same as [KCl] added. Because of the difference in volumes between intracellular and extracellular spaces in the local environment, some KCl will exist excess in the extracellular environment, as charge-balanced pairs, and only a fraction of K^+ equilibrates across the membrane, which leaves only a fraction of unbalanced extracellular Cl^-. This ratio can be calculated. From microscopy images, we find that the intracellular area of cells corresponds to 362816 px out of the total 1024x1024 px image (mean, n=3, raw images not shown). Using these values as a proxy gives:

$$\frac{Intracellular\ Volume}{Extracellular\ Volume} = \frac{362816}{1024^2 - 362816} = 0.529$$

This is not far off from the expected ratio of volumes of perfect spheres packing in space, ~0.64. Incorporating this correction factor into the equation for ΔV_m gives:

$$\Delta V_m = \frac{RT}{zF} ln(1 + \frac{[KCl]_{added} \text{x } 0.529}{0.000624})$$

Equation 2: Calculation of ΔV_m based on addition of [KCl] in presence of 10 μM valinomycin.

Thus, we can convert values for [KCl] added into expected ΔVm in the presence of valinomycin, based on the excess extracellular negative charge of Cl^- ions.

Table S4: [KCl] concentrations and their expected effect on ΔV_m, calculated according to Equation 2.

[KCl] added (mM)	Calculated ΔV_m (mV)
0	0
1	1.577
10	5.777
50	9.686
100	11.437
150	12.468
500	15.548

We next tested a titration of KCl into cells pretreated with valinomycin (1 hr), and quantified their intracellular ThT concentrations 20 mins after KCl addition (Figure S19).

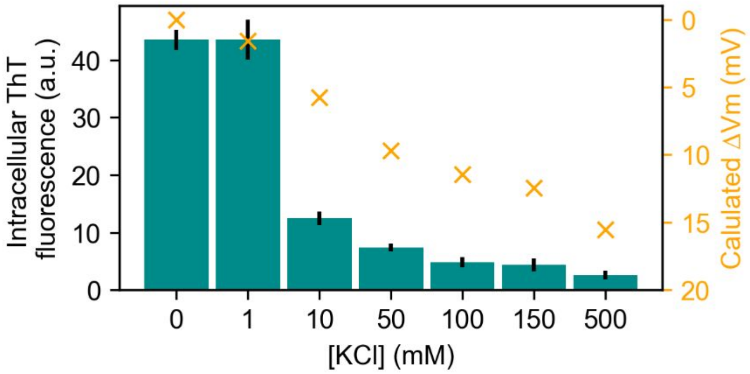

Figure S24. Intracellular ThT fluorescence calculated in *Synechocystis* cells pretreated with 10 μM valinomycin and increasing concentrations of [KCl]. Calculated V_m from Table S4.

From this we can see that the trend of change in intracellular ThT fluorescence is similar to the calculated ΔV_m but quantitatively they do not provide a good fit. This is most likely because during equilibration cells are actively pumping K+ and/or Cl- ions (i.e. regulating their V_m), so the calculated ΔV_m is not representative of reality. This means that accurately and definitively calibrating intracellular [ThT] relative to actual V_m is technically challenging and was not used in this study.

References:

(1) Rippka, R.; Deruelles, J.; Waterbury, J. B.; Herdman, M.; Stanier, R. Y. Generic Assignments, Strain Histories and Properties of Pure Cultures of Cyanobacteria. *Microbiology* **1979**, *111* (1), 1–61. https://doi.org/10.1099/00221287-111-1-1.

(2) Yang, C.-C.; Wen, R. C.; Shen, C. R.; Yao, D.-J. Using a Microfluidic Gradient Generator to Characterize BG-11 Medium for the Growth of Cyanobacteria Synechococcus Elongatus PCC7942. *Micromachines* **2015**, *6* (11), 1755–1767. https://doi.org/10.3390/mi6111454.

(3) Lea-Smith, D. J.; Ross, N.; Zori, M.; Bendall, D. S.; Dennis, J. S.; Scott, S. A.; Smith, A. G.; Howe, C. J. Thylakoid Terminal Oxidases Are Essential for the Cyanobacterium Synechocystis Sp. PCC 6803 to Survive Rapidly Changing Light Intensities. *Plant Physiol.* **2013**, *162* (1), 484–495. https://doi.org/10.1104/pp.112.210260.

(4) Ehrenberg, B.; Montana, V.; Wei, M. D.; Wuskell, J. P.; Loew, L. M. Membrane Potential Can Be Determined in Individual Cells from the Nernstian Distribution of Cationic Dyes. *Biophys. J.* **1988**, *53* (5), 785–794. https://doi.org/10.1016/S0006-3495(88)83158-8.

(5) de Souza-Guerreiro, T. C.; Bondelli, G.; Grobas, I.; Donini, S.; Sesti, V.; Bertarelli, C.; Lanzani, G.; Asally, M.; Paternò, G. M. Membrane Targeted Azobenzene Drives Optical Modulation of Bacterial Membrane Potential. *Adv. Sci.* **2023**, *10* (8), 2205007. https://doi.org/10.1002/advs.202205007.

(6) Han, J.; Burgess, K. Fluorescent Indicators for Intracellular pH. *Chem. Rev.* **2010**, *110* (5), 2709–2728. https://doi.org/10.1021/cr900249z.

(7) Mangan, N. M.; Flamholz, A.; Hood, R. D.; Milo, R.; Savage, D. F. pH Determines the Energetic Efficiency of the Cyanobacterial CO2 Concentrating Mechanism. *Proc. Natl. Acad. Sci.* **2016**, *113* (36), E5354–E5362. https://doi.org/10.1073/pnas.1525145113.

(8) Clerc, S.; Barenholz, Y. A Quantitative Model for Using Acridine Orange as a Transmembrane pH Gradient Probe. *Anal. Biochem.* **1998**, *259* (1), 104–111. https://doi.org/10.1006/abio.1998.2639.

(9) Teuber, M.; Rögner, M.; Berry, S. Fluorescent Probes for Non-Invasive Bioenergetic Studies of Whole Cyanobacterial Cells. *Biochim. Biophys. Acta BBA - Bioenerg.* **2001**, *1506* (1), 31–46. https://doi.org/10.1016/S0005-2728(01)00178-5.

(10) Cohen, A. E.; Venkatachalam, V. Bringing Bioelectricity to Light. *Annu. Rev. Biophys.* **2014**, *43* (Volume 43, 2014), 211–232. https://doi.org/10.1146/annurev-biophys-051013-022717.
